# High performance work system and innovative work behaviour: A moderated mediation analysis of knowledge sharing and employee creativity in Nigerian higher education institutions

**DOI:** 10.1371/journal.pone.0338031

**Published:** 2025-12-31

**Authors:** Azadeh Amoozegar, Oghenekevwe Esohwode, Mohammad Falahat, Hariharan N. Krishnasamy, Temoor Anjum, Valliappan Raju, Mohammad Ali Hasan

**Affiliations:** 1 Faculty of Education and Liberal Arts, INTI International University, Persiaran Perdana BBN Putra Nilai, Nilai, Negeri Sembilan, Malaysia; 2 Limkokwing Graduate School, Limkokwing University of Creative Technology, Inovasi 1-1, Jalan Teknokrat Cyberjaya, Selangor, Malaysia; 3 Strategic Research Institute (SRI), Asia Pacific University of Technology and Innovation (APU), Jalan Teknologi 5, Taman Teknologi Malaysia, Wilayah Persekutuan Kuala Lumpur, Malaysia; 4 Faculty of Education and Liberal Arts, INTI International University, Persiaran Perdana BBN Putra Nilai, Nilai, Negeri Sembilan, Malaysia; 5 University Institute of Management Sciences, PMAS-Arid Agriculture University Rawalpindi, Shamsabad, Rawalpindi, Pakistan; 6 Business Department, GISMA University of Applied Sciences, Berlin, Germany; 7 Faculty of Arts and Science, American University of Science, 50 Truong Dinh, Districy 3, Ho Chi Minh City, Vietnam; Sichuan University, CHINA

## Abstract

An examination of the literature on human resource management (HRM) and innovation reveals a lack of understanding regarding the mechanism through which HRM and innovation are connected. To address this, the present study proposes a moderated mediation model to elucidate the relationship between HRM, specifically its novel High Performance Work Systems (HPWS), and innovative work behaviour (IWB). This model suggests that employee creativity serves as a mediator between HPWS and innovative work behaviour. Furthermore, the proposed framework incorporates the moderating influence of knowledge sharing on the relationships between HPWS, employee creativity, and innovative work behaviour. The study employed a quantitative methodology with a cross-sectional design, focusing on academic staff at federal and state universities in Nigeria. A self-administered questionnaire was utilised to gather data from 307 employees. The statistical analysis was conducted using SmartPLS 4 software. The findings demonstrated that employee creativity served as a partial mediator in the association between HPWS and IWB, explaining 75.6% of the variance. Furthermore, the study confirmed that knowledge sharing moderated the influence of HPWS on both employee creativity and IWB. The research proposed a novel explanation for the connection between HPWS and innovative work behaviour, addressing the identified gap that potentially involves numerous variables acting as intermediaries or influencing factors. This suggests the need for further exploration to uncover additional potential mechanisms.

## 1. Introduction

In response to mounting challenges from globalization, shifts in higher education funding models, and fluctuating supply and demand dynamics, numerous higher education institutions worldwide are striving to remain viable and gain competitive edges through innovative approaches [1]. Furthermore, in the contemporary era of digitalisation and globalisation, the demand for employees possessing creativity and innovative capabilities as fundamental attributes is becoming increasingly prevalent [2]. Organizations that fail to embrace innovation risk a gradual decline leading to their eventual collapse [3]. The key to an organization’s long-term success lies in its ability to innovate [4]. This is particularly vital in rapidly dynamic markets like education [3]. The dynamic nature of the education field necessitates that universities, as hubs of knowledge, promote innovative work practices among their employees to excel in the current environment [5]. An example of this is the restructuring of courses and curricula, along with the implementation of innovative educational approaches [6]. This dynamic landscape demands that employees adopt innovative methods and initiatives to effectively fulfil their job responsibilities [5]. However, while there is an increasing focus on corporate innovation, limited research exists on methods to encourage individual-level innovation within organizations [7].

Nigeria has the largest higher education sector in Africa, accommodating millions of students across numerous institutions. However, despite this substantial enrolment, the quality of education offered by Nigerian universities has significantly declined in recent years [8]. This decline is deeply linked to inadequate funding, insufficient lecturers, poor infrastructural facilities, an unstable academic calendar, academic corruption, insecurity, brain drain, weak leadership, poor research output, and limited staff development, all of which profoundly affect innovation [8]. In this respect, Odiaka [9] suggested that research examining the understanding of high-performance work practices, policies and guidelines across organisations and industries would be informative in Nigeria. Therefore, adopting HPWS tailored to the unique challenges of Nigerian HEIs is critical to rebuilding academic staff capacity, enhancing innovation, and ultimately improving educational quality and performance. Furthermore, considering the economic and social challenges Nigeria currently faces—including political instability, corruption, high unemployment, and gender inequality—it is essential to prioritize the education sector as a key driver for sustainable economic growth.

Innovative behaviour involves the deliberate implementation, rather than just the creation, of new and beneficial ideas [10]. Innovative behaviour encompasses the methods and steps involved in creating, sharing, applying, and actualizing novel ideas [7]. Innovation is a key element of organizational strategy, essential for encouraging creative behaviour among employees and promoting the growth of productivity, market advantage, and sustainable success of the enterprise [11]. Moreover, an organization’s ability to endure in the face of growing industry rivalry depends on the adoption of innovative methods [12]. Innovative behaviour is adopted by employees as a tactical method to realize organizational aims and goals. This encompasses the creation, handling, and execution of novel concepts that boost the organization’s competitive advantage and long-term sustainability. Such deliberate actions contribute to the organization’s sustainability and market advantage [13]. In this context, global researchers have devoted considerable focus to methods for achieving innovation [14–16]. However, a significant portion of this research has been directed by business models that aim to foster innovation through the exploration of advantageous combinations of technology, knowledge, and science, as well as by examining how these models can be applied and the insights that can be gained from them [17]. Importantly, beyond business models focused on technology and knowledge, human resources (HR) play a vital role in influencing innovative performance. According to the resource-based view theory, HR is a significant internal asset that shapes intangible skills and identifies both innovation capabilities and the competitiveness of a firm [18].

Universities and colleges play a vital role in spearheading and directing innovation [19]. Tri, Nga [3] found that innovation is a key element in securing sustainable competitive advantages, which are essential for adapting to a swiftly evolving business environment. Numerous researchers have verified that employee-generated creative ideas are a key component of organizational creativity, serving as the primary source of innovation [20]. Innovative work behaviour encompasses the generation of novel and beneficial ideas for products, services, processes, and procedures [21]. However, creativity is recognized as a vital element linked to innovation, as it involves the application of ideas within innovative work behaviour [22]. Tri, Nga [3], highlight that creativity serves as the cornerstone for generating innovative concepts, indicating that creative thinking initiates the innovation process. According to O’Regan, Ghobadian [23] the creativity of individual employees plays a key role in driving innovation within an organization, as it is one of the most significant factors influencing organizational innovation. Yuan and Xie [24] stated that fostering employee creativity is important for enhancing organizational innovation and gaining a competitive edge. Although creativity serves as the foundation for innovation by generating novel ideas that result in innovative outcomes, there is a scarcity of empirical research examining creativity as a mediating factor between external influences and innovation [3,25]. Consequently, these pathways lay the groundwork for understanding how connections are established.

When considering innovation in higher education, the faculty’s contribution assumes an exceptionally critical nature. The faculty, being the primary catalysts within universities, assume a pivotal and strategic position in a multitude of scholarly and research endeavours [19]. The reputation of the institution and the quality of education are both immediately impacted by the performance of its faculty, which is increasingly defined by their degree of ingenuity and innovative conduct [26]. The innovative conduct of academic staff is highly beneficial, as it can result in both economic and psychological advantages through favourable changes in administrative, technological, or social aspects of the organization’s current condition [10]. Kim, Nurunnabi [27] also maintain that employee conduct can facilitate the examination of organizational-level outcomes, as it embodies an individual’s attitudes and behaviours. As a result, their mindsets, views, and actions represent the critical edge of tangible outcomes at the organizational level [17]. While innovative work behaviour (IWB) among academic staff is essential and has attracted worldwide research attention, there is a significant scarcity of research resources on this subject in Nigeria. Therefore, it is imperative to pinpoint the elements that can enhance IWB, given its critical role as a primary contributor to an organization’s competitive advantage [28].

The process of innovation is not a straightforward conversion of research and development (R&D) results into successful outcomes. In reality, it involves a complex interplay of various factors beyond just technology and knowledge [17]. Existing literature has identified several influential elements, with high performance work system emerging as a significant predictor of employees’ innovative work behaviour (IWB) [17,29]. High performance work system encompasses HR strategies that collectively create a positive work environment, enhancing employees’ capabilities, motivation, and growth opportunities [29]. Furthermore, it facilitates improved information exchange and sharing among employees, thereby fostering the generation of creative ideas and perspectives [24]. Al-Ajlouni [17] and Shin, Jeong [30] highlighted the need for additional research on the relationship between high-performance work system and innovative work behaviour. To address this research gap identified by previous scholars, this study proposes a moderated mediation mechanism to elucidate the connection between high-performance work systems (HPWS) and innovative work behaviour in the specific context of higher education.

Although previous studies have enhanced our understanding of the elements influencing innovative work behaviour, it is critical to identify several significant gaps in the existing literature before drawing firm conclusions. First of all, most existing studies on HPWS and innovative work behaviour primarily focus on corporate environments, leaving a significant gap in understanding how these relationships manifest among academic faculty members within Nigerian higher education institutions (HEIs). The distinct cultural, institutional, and resource-related challenges in Nigerian HEIs create a unique context that shapes human resource management practices and influences innovative work behaviours differently from corporate settings. This contextual difference highlights the need for targeted research within Nigerian academia to better capture these dynamics and their implications. Secondly, although previous studies have established a link between HPWS and IWB, there remains a significant gap in research exploring the mediating and moderating effects of employee creativity and knowledge sharing [1] in the relationships between HPWS and creativity, as well as HPWS and IWB. Enhancing HPWS is believed to foster various positive behaviours among employees, including increased creativity [17]. Thirdly, the majority of research on high-performance work systems (HPWS) is predominantly based on data from the American-European context [31]. Empirical investigations into how HPWS influence employee creativity and innovation in countries outside this mainstream, such as Nigeria, are limited [9].

This research seeks to make significant contributions to the existing literature in at least three key areas. Firstly, it has been noted by scholars that developing and implementing human resources management (HRM) practices, such as high performance work system are decisive and play a key role in improving employee outcomes [2]. Most of the existing studies in this area have been conducted within small and medium-sized enterprises (SMEs) [17,32–34], hospitality sector [35], manufacturing [36–38], telecommunication sector Jyoti and Rani [39], and firms [40,41]. Only Escribá-Carda, Balbastre-Benavent [42], Alsafadi, Al-Okaily [43] empirically examined relationships between HPWS and innovative behaviours in the higher education institutions sector. Whilst numerous scholars have made significant contributions to understanding the link between high performance work systems and employee outcomes, many have focused on creativity as the final variable, overlooking the importance of innovative work behaviour [24,34,41]. Second, this study broadens the scope of research on interactive patterns predicting innovative work behaviour by investigating the presence of employee creativity mediating effect of HPWS. Specifically, this research offers a perspective on approach and avoidance regarding the positive primary and combined effects of HPWS on innovative behaviour, thereby enhancing academic comprehension of how employee creativity can specifically enhance innovative actions in the workplace. Third, this study adds to the growing body of research on the positive links between HPWS, employee creativity, and innovative work behaviour by examining whether knowledge sharing positively influences this relationship. The insights obtained from this study can be utilized to enhance HR policies and frameworks, which outline innovative methods, facilitators, interventions, and recommendations to encourage IWB, particularly within the HEIs sector.

## 2. Hypothesis development and conceptual framework

**Fig 1:**
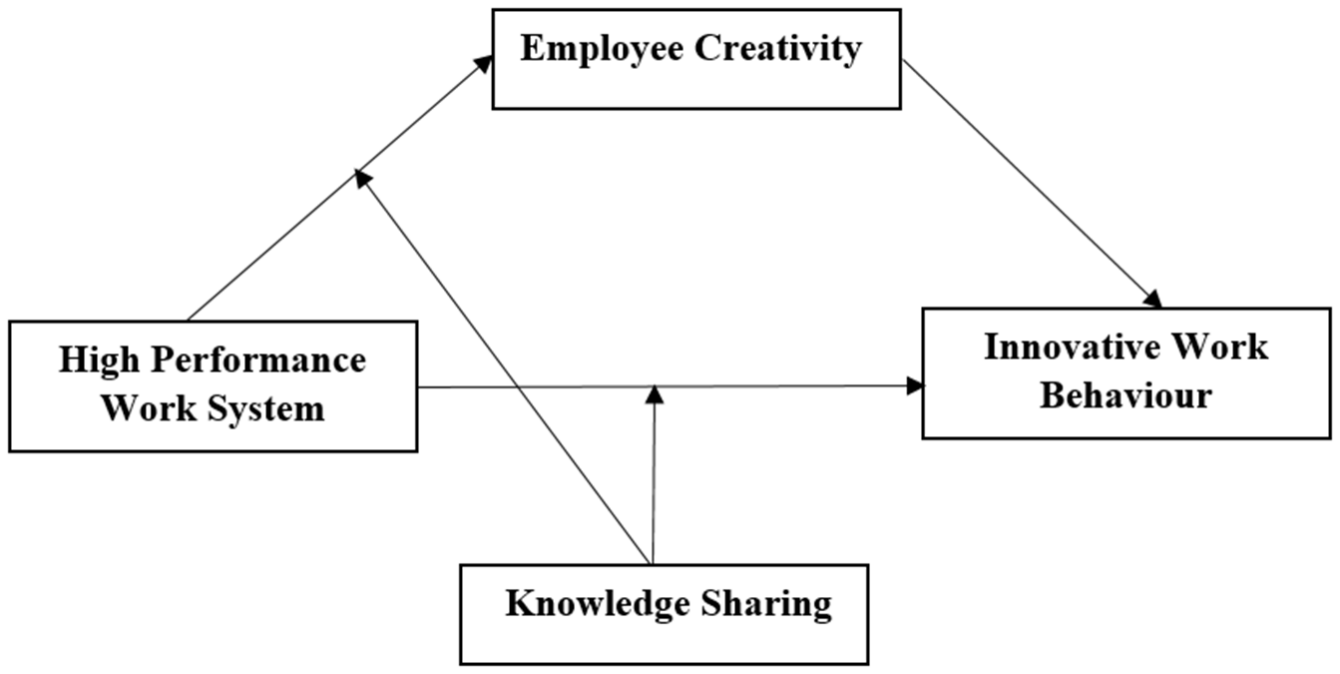
Conceptual model.

Fig 1 illustrates the conceptual model. The model posits that high performance work system (HPWS) can directly promote innovative work behaviour. The model also predicts that HPWS impose direct significant impact on employee creativity. Additionally, the effect of employee creativity on innovative work behaviour is positive and significant. Furthermore, the link between HPWS and IWB is mediated by employee creativity. Finally, the model suggests that the sharing of knowledge moderate the connection between HPWS and both employee creativity and innovative work behaviour.

### 2.1 Innovative Work Behaviour (IWB)

Innovative work behaviour (IWB) plays a significant role in driving organizational growth in the current era of globalization [44]. Despite the efforts of various scholars to define innovative work behaviour [4,45,46], the definition most commonly referenced in the literature is offered by Janssen [47], who defined IWB as the purposeful act of creating, introducing, and applying new ideas within a work role, team, or organization, with the intention of enhancing the performance of the role, team, or organization. Innovative work behaviour includes a variety of actions, such as generating new ideas, promotion of these ideas and assisting in their implementation [48]. As stated by Tri, Nga [3], innovative work behavior involves an individual’s efforts to initiate and intentionally introduce new and advantageous ideas, processes, products, or procedures within their job. Numerous studies have demonstrated the key role of innovative behavior in enhancing business performance, efficiency, and competitive edge [49,50]. Consequently, the education sector also needs to develop and implement creative strategies to stay competitive by introducing novel concepts and practices [44].

Innovative work behavior (IWB) inspired employees to introduce novel and atypical ideas, strategies, and viewpoints [44]. Employees possess the expertise and capabilities to recognise challenges and develop innovative solutions in the workplace within their work environment. A substantial body of research on employee-driven innovation demonstrates how workplace innovation can effectively enhance a company’s performance [51]. Within the realm of education, IWB encompasses the efforts of educational professionals to create, advocate for, and implement innovative concepts, methodologies, or resources that aim to enhance the quality of instruction, learning experiences, or the broader educational setting. This staff competence is vital in boosting the overall quality of service and performance, which are essential for an organisation’s competitive advantage, prosperity, and longevity [2]. It involves transcending conventional practices and pursuing imaginative answers to educational challenges. In spite of the importance of IWB for creating positive organizations and remaining competitive, this emerging research field remains underexplored [2]. Consequently, there are numerous appeals to expand the current body of knowledge in this area [29], particularly in Africa. However, there has been a scarcity of studies examining the factors influencing innovative work behaviors within the education sector up to now [44,52].

The second stage of the creative-innovative process, known as innovation, entails the development of a new method or product [53]. Asurakkody and Kim [54] characterize innovative work behaviour as a multi-stage process involving the generation of ideas, exploration of opportunities, championing those ideas, and putting them into practice, ultimately leading to new products. Since innovation is rooted in the creation and cultivation of ideas, it is crucial to explore the factors that foster innovative work behaviour [42]. Nevertheless, research on innovation faces challenges in pinpointing key factors due to the intricate nature of diverse contexts, organizational hierarchies, and processes linked to innovative practices [53]. Given the practical challenges involved, researchers have suggested classifying innovation according to different levels of analysis, including organizational, individual, and team levels. In this context, scholars have proposed various factors that contribute to innovation. These include high performance work practices [2], transformational leadership [55], workplace happiness and work engagement [56], perceived creativity [57], organizational climate, psychological empowerment, and high performance work system [29], and knowledge sharing [54]. At the individual level, the principles of social exchange theory imply that HRM promotes discretionary behaviours that support innovation [42]. Yet, there remains a gap in comprehensive understanding of how to nurture innovation at the individual level [45].

### 2.2 The link between high-performance work systems (HPWS) and innovative work behaviour

Over the past three decades, research in psychology and management has focused on understanding how HRM practices influence outcomes for organizations and employees, including aspects like business performance, productivity, innovation, and employee performance [45]. Some researchers concentrate on HRM practice frameworks, such as high-performance work systems [39,58]. A high-performance work system is a set of human resource management practices aimed at enhancing employees’ skills, motivation and engagement, thereby enabling an organisation to achieve a lasting competitive edge in the market [41]. High-performance work systems (HPWS) are essential as they represent the strategic philosophy and practices of human resource management [59]. HPWS encompass a set of strategies aimed at achieving outstanding performance from individuals [60]. This involves a distinctive blend of training initiatives, organizational structures, and processes that improve employees’ awareness of their abilities, enhance their innovative potential, and motivate their active participation in work and decision-making activities [43]. High-performance work systems emphasize employee participation and are committed to fostering an organizational culture that focuses on commitment rather than control [61]. High performance work systems refer to a system that establishes a work environment enabling employees to have more involvement and accountability [62]. HPWS facilitates knowledge sharing among employees and encourages the development of innovative ideas [43]. Organizations that implement HPWS tend to invest more in the development and progression of their employees. This involves catering to their ambitions for career growth and personal development [63].

Current scholarly works have emphasised the significance of novel integrated human resource management (HRM) practices incorporating high-performance work systems (HPWS). The high-performance work system as the latest form of strategic human resource management, has been extensively examined by scholars [32]. This focus stems from the key role these practices play in enhancing employee productivity and boosting organisational performance [17,43]. By aligning HR practices to regard employees as esteemed owners and collaborators, organizations can enhance value and bolster their chances of achieving and sustaining a competitive edge [64]. High-performance work systems (HPWS) can facilitate the exchange of information amongst staff employees, thereby encouraging them to develop innovative ideas [65]. Research has indicated that organisations implementing HPWS are more inclined to invest in their workforce, value employee involvement in decision-making processes, and address staff members’ aspirations for career advancement and personal growth [41]. [32] suggested that employee innovative behaviour can be effectively enhanced through high-performance work systems. Khawaldeh and Alzghoul [66] posit that the inherent elements of HPWS, namely adaptability and flexibility, contribute to creating an atmosphere that welcomes creative ideas and innovative methods, thus encouraging their growth and progression. Nonetheless, there are only a limited number of empirical studies that have examined the link between HRM practices and organizational innovation, with even fewer exploring innovative work behaviour [42,45]. Consequently, this research hypothesises that a high-performance work system characterised by its systematic approach and comprehensive nature is likely to foster and encourage innovative behaviour among employees.

Current research emphasises that innovation is the responsibility of every member within an organisation, as the success of a company is determined by the attitudes and behaviours of its workforce. Consequently, high-performance work systems (HPWS) must address different aspects that impact employee performance, with a particular focus on fostering creativity [17]. This encompasses the development of interpersonal adaptability, effective communication skills, and cultural awareness, alongside the organisation’s commitment to investing in training programmes, educational initiatives, and technological advancements [67]. Jiang, Wang [68] suggest that this can be accomplished by recruiting highly skilled employees, providing them with training and knowledge resources, while simultaneously maintaining reward structures and communication channels that foster a more motivating work environment. This approach, implemented through HPWS, ultimately encourages employees and promotes their creative behaviours [17]. The social exchange theory expands upon these concepts and reinforces the significant link between HRM and creativity. Social Exchange Theory (SET) suggests that high-performance work systems (HPWS) lead to better employee outcomes because when employees sense that they are valued by their organization, they tend to respond with favourable attitudes and behaviours [35]. This conclusion is further corroborated by various research studies, including those conducted by Alsafadi, Al-Okaily [43], Chiang, Hsu [69], Rasheed, Shahzad [70] and Tang, Yu [41]. Nonetheless, the literature emphasises examining how HPWSs impact employee behaviour. In accordance with our research objectives, this investigation centred on how perceptions of HPWSs influence specific behaviours, namely innovative work behaviour (IWB). Considering the arguments in the above paragraphs, this study put forward the following hypotheses:

*H1:* High performance work system (HPWS) significantly affect innovative work behaviour.

### 2.3 The link between high-performance work systems (HPWS) and employee creativity

High-performance work systems (HPWS) have been widely recognized as an effective approach to enhancing organizational performance [33]. According to Chan and Chu [71], high-performance work systems (HPWS) comprise a range of integrated human resource management practices designed to improve performance outcomes at both the employee and organizational levels. The influence of HPWS on organizational outcomes is best understood through its impact on individual employee perceptions, which in turn shape their attitudes and behaviours. Among the positive individual outcomes associated with HPWS, enhanced employee creativity stands out as a significant contributor to overall organizational success [72]. The relationship between high-performance work systems and employee creativity is understood as an exchange process in which employees receive preferred human resource management practices—such as HPWS—from their organization, which consequently encourages them to enhance their creativity [17]. While the impact of high-performance work systems (HPWS) on creativity is widely acknowledged, there remains a notable gap in comprehensive research exploring how human resource systems influence employee creative behavior [71]. Escribá-Carda, Balbastre-Benavent [42] emphasize the importance of investigating the underlying mechanisms linking HPWS and employee creativity in order to better understand these processes and to optimize HR practices that foster innovation.

The literature on high-performance work systems (HPWS) often explicates the positive effects of HPWS on employee outcomes through the lens of social exchange theory [73]. Employees who perceive benefits from the high-performance work systems (HPWS) implemented by their organizations often feel a responsibility to reciprocate by increasing their effort and dedication [17]. In doing so, they are motivated to engage in creative behaviors, such as investing additional effort in understanding problems, seeking solutions, and generating a wide range of alternatives [74]. Complementing this perspective, several studies employ the Ability-Motivation-Opportunity (AMO) framework to argue that HPWS equips employees with the essential abilities, motivation, and opportunities needed to foster creativity [33,41]. In essence, HPWS fosters employees’ intrinsic motivation, autonomy, and relevant job-related skills, which theoretically lead to enhanced creative performance [74]. High-performance work systems (HPWS) represent a strategic approach that leverages the strengths of organizational management to enhance employees’ capabilities in generating diverse and creative ideas [34]. Hence, HPWS is widely conceptualized as a valuable job resource that nurtures and promotes employee creativity [72].

*H2:* High performance work system (HPWS) significantly affect employee creativity.

### 2.4 Employee creativity as a mediator of HPWS-innovative work behaviour relationship

In organizations, creativity is often seen as the production of new and practical ideas [48]. Creativity pertains to the cognitive and behavioral actions applied when attempting to implement novel ideas [53]. Creativity can be defined as the ability to generate effective solutions to challenges or to conceive innovative and engaging ideas across various fields, which result in the designing the products and/or artifacts and influence thinking [75]. The concept of employee creativity refers to the ability of workers to come up with innovative and meaningful ideas within the organization [24]. Creative professionals tend to excel in producing innovative and novel ideas, often surpassing their competitors [22]. Creativity requires curiosity and imagination to gain new insights, merge resources, and devise innovative approaches to meet unfulfilled market demands [76]. Creative individuals effectively utilize cutting-edge information technologies, demonstrate creative thinking, and apply inventive approaches when tackling complex challenges in their professional or personal lives [25]. As a result, enhancing employees’ creative potential is an essential element in achieving organizational success [77].

In universities, the creativity of faculty members is essential due to worldwide progress [43]. It represents the conception of innovative and valuable ideas, resulting in the development of new products and services that enhance university advancement, sustainable growth, and competitive advantage [78]. A faculty member’s creativity is characterized by their capacity to produce novel and valuable concepts, which significantly impacts organizational innovation [21,79] or innovation within a university or company [80]. As employee creativity grows, it is anticipated that the innovative ideas and clever solutions they generate will boost their efficiency and performance levels [25]. Given that employees are a vital source of innovation, the challenge lies in how to encourage innovative work behavior (IWB), which refers to employees’ proactive approach in creating and implementing new ideas at work [81]. Moreover, the duty of a creative faculty member is to nurture their students’ creativity by employing modern and innovative methods to impart scientific knowledge and information [82]. It enables students to explore various approaches to foster an environment conducive to creativity and to introduce contemporary innovative concepts [83]. Recognizing the importance of a faculty member’s creative abilities, several studies have explored the factors and influences that contribute to these processes [17,53].

The ongoing success of organizations is predicated on their continuous capacity for innovation. Yet, what researchers identify as innovation is actually the outcome of two interconnected elements: creativity and its implementation [84]. Creativity and innovative behaviour are frequently connected because scholars often perceive them as the initial and final stages of a multi-step process, or they regard them as closely related [2]. In spite of this commonly recognized viewpoint and the interactionist model of creativity, a growing body of scholars challenges this simplistic view, emphasizing a more complex relationship and arguing that creativity fundamentally differs from innovation [2]. According to Amabile [85], creativity involves generating new and valuable ideas, while innovation is described as the process of refining and applying these ideas to ensure they are practical and usable. Creativity forms the basis for innovation while innovation can be seen as the effective implementation of creative ideas within an organization [86]. There is a general consensus that creativity is essential for fostering a higher level of innovative behavior within an organization [85,87]. Chaubey, Sahoo [88] suggested that innovation and creativity are interconnected processes. The emergence of a valuable idea often paves the way for innovative advancements. Individuals who are able to come up with novel, new, and practical ideas, they are more likely to be innovative, which subsequently enhances innovation within groups and organizations [89]. Tri, Nga [3] stated that creativity is considered a key element for fostering innovative work behaviour. In a similar vein, Yuan and Xie [24] found that employee creativity plays a significant role in driving organizational innovation. Although employee creativity is fundamental to the innovative work behavior (IWB) model, as it transforms ideas into practical workplace enhancements, there is limited research examining whether creativity can boost IWB, particularly in educational settings [90]. Nevertheless, the progression of studies in this field has demonstrated an ongoing interest in uncovering the antecedents and influences linked to creativity and innovation [53].

In the previous section, the researcher established that HPWSs have a direct influence on employee creativity and innovative work behaviour. High-performance work systems (HPWS) that emphasise employee investment, provide opportunities for growth, and encourage participation in organisational decision-making processes lead workers to feel supported and consider themselves integral to a reciprocal social exchange relationship [24]. This perceived support often results in employees exhibiting a more positive demeanour, which in turn facilitates and nurtures their creative abilities. Research conducted by Huang, Sardeshmukh [91] on teachers in Chinese public schools revealed a favourable correlation between high-performance work systems (HPWS) and the creativity of teachers. Similarly, Al-Ajlouni [17] examined the influence of HPWS on employee creativity and organizational innovation. Nevertheless, research utilising employee creativity as a mediating factor remains scarce [25,92]. Although these investigations have shown innovation outcomes at the organizational level, they have not thoroughly explored the specific impacts at individual level [53]. Drawing from the concepts of the dynamic componential model of individual creativity [22], which suggests that innovative work behaviour (IWB) is dependent on an individual’s level of creative cognitive abilities, and in line with the Job Demands-Resources (JD-R) model [2,93], creativity is considered a personal resource that employees can utilise to demonstrate innovative behaviour. Consequently, this research hypothesised that the extent and regularity of IWB would be further enhanced when a creative employee is supported by high-performance work systems (HPWSs). Thus, employee creativity was proposed as a mediating factor in the relationship between HPWS and IWB. Therefore, the following hypothesis has been formulated:

*H3:* Employee creativity significantly affect innovative work behaviour.

*H4:* Employee creativity mediate the relationship between high performance work system (HPWS) and innovative work behaviour.

### 2.5 Knowledge sharing attribution as a moderator

Knowledge sharing has emerged as one of the most significant research subjects in management [94]. Knowledge serves as a strategic asset of organizations that should be disseminated throughout the company to be effectively leveraged as a competitive advantage [95]. Knowledge sharing involves a series of deliberate actions where individuals exchange data or significant knowledge to collaborate with others in the creation of innovative ideas and the implementation of policies [54]. Similarly, knowledge sharing is a process of sharing information and data which can enhance workplace performance by improving employees’ abilities in areas such as learning, decision-making and problem-solving [96]. According to Khawaldeh and Alzghoul [66], knowledge sharing refers to various methods and initiatives that enable the exchange of information, skills, and deep insights amongst members of an organisation. This exchange subsequently promotes learning and cultivates innovative ideas. Broadly speaking, the sharing of knowledge encompasses the exchange of experiences and organizational insights related to business operations through communication channels among individuals [97]. Hence, organizations that encourage and support their staff in sharing knowledge within teams and across the organisation are likely to foster the creativity and stimulate new business prospects. This, in turn, can drive organisational innovation initiatives [98].

Universities and colleges serve as venues for scholars to exchange ideas and knowledge [99]. According to Sallis and Jones [100] academics are skilled professionals who generate, utilise, disseminate, and implement knowledge through activities such as instruction and scholarly inquiry. Educational institutions inherently foster knowledge sharing to a degree unmatched by other professions, potentially yielding diverse results, which include the creation of protocols, involvement in research committee, conferences and publications, scientific discussions, and the production of documents, as well as the enhancement of expertise (Asurakkody & Kim, 2020). When employees exchange information with their colleagues, it expands the collective knowledge of the workforce and enhances the potential for generating innovative concepts [101]. The knowledge that is generated and stored will act as a resource to aid scholars and researchers in advancing the cycle of knowledge and enhancing the institution’s standing in the academic marketplace [102]. The influence of knowledge sharing in these settings may surpass that of business organizations [103]. Therefore, to gain a competitive advantage and improve their performance, HEIs must formulate strategies that use the knowledge that academics possess [95].

Previous studies have largely overlooked the impact of knowledge sharing on individual creativity and innovation [104]. Knowledge sharing is essential for both the creation and implementation of organizational knowledge, which are key components in driving innovation within a company [94]. Kremer, Villamor [105] stated that knowledge sharing is a critical factor that promote innovation. According to Belso-Martinez and Diez-Vial [106], companies that enhance their participation in knowledge sharing generally experience an improvement in their ability to innovate. Knowledge sharing fosters communication and builds mutual trust among employees as they share their experiences and knowledge, which positively influences employees’ IWB and boosts organizational performance [107]. Faris Hussain, Hanifah [108] highlighted the importance of individual knowledge sharing to foster innovative behavior. Empirical studies generally support a positive link between knowledge sharing (KS) and employees’ innovative work behavior (IWB) [58,109]. Consequently, gaining knowledge and skills through collaboration has proven to be an effective and efficient approach to achieving successful innovation [94].

The realization of creativity requires various resources, including time, materials, collaborative efforts, substantial labour, knowledge sources, and intense cognitive exertion. Among these, knowledge stands out as a fundamental resource that enhances individual creative abilities [104]. Amabile [85] highlights the vital role that knowledge plays in fostering creativity among employees. According to Lee [104], knowledge sharing among organizational members is essential for fostering individual creativity, as it facilitates collaboration and enhances domain expertise within the organization [110]. Knowledge sharing promotes the circulation of knowledge, combines recognition resources, and encourages divergent thinking and novel ideas. This process also conserves significant time and resources, all of which are essential for fostering creative performance [40]. Lee [104] discovered a positive correlation between the degree of knowledge-sharing among Korean students in higher education institutions and their personal creative outcomes. According to Kim and Park [111], it is important for employees to exchange their knowledge at work because it enhances their creativity. Maulding [53] argued that knowledge sharing plays fundamental role in support of creativity within organization settings. Hence, employees within an organization must consistently depend on their colleagues’ knowledge (expertise and experience) or utilize the available explicit information within the organization to tackle new tasks and sustain their creativity [112].

A potential key advantage of HR systems may be the fostering of an environment that encourages the exchange of knowledge and the sharing of ideas and information during interactive processes [40]. The body of existing research consistently affirms that high-performance work systems (HPWS) significantly influence the process of knowledge sharing. For instance, Despita, Yuliani [113], Almadana, Suharnomo [114], and Abbasi, Shabbir [115] have highlighted the significant role of HPWS in promoting knowledge sharing in organizational environments. Chuang, Jackson [116] similarly observed that HRM systems functioned as stimuli to foster knowledge-sharing behaviours. HPWS plays a role in enhancing trust and encouraging community involvement, which in turn increases the likelihood of knowledge dissemination [66]. Further, HPWS involves the incorporation of recognition and reward systems that are strategically crafted to foster a culture of appreciation and motivation, with an emphasis on encouraging employees to share knowledge [82,117]. The effectiveness of High-Performance Work Systems (HPWS) in stimulating employee creativity and innovative work behavior (IWB) is enhanced when an organization actively encourages knowledge sharing. Consequently, this research proposes that knowledge sharing acts as a moderator, strengthening the positive link between HPWS and both employee creativity and IWB among academic personnel. This leads to the formulation of the following hypothesis:

*H5:* Knowledge sharing moderates the effect of HPWS on IWB. This relationship is stronger when knowledge sharing is greater.

*H6:* Knowledge sharing moderates the effect of HPWS on employee creativity. This relationship is stronger when knowledge sharing is greater.

## 3. Methodology

### 3.1 Design and procedure

According to Bell, Harley [118] and Alnehabi and Al-Mekhlafi [119], the steps in quantitative research can follow a straightforward path from developing a theory to obtaining results. This research employed a quantitative cross-sectional approach to investigate the connections among high-performance work systems, employee creativity, innovative work behavior, and knowledge sharing. The study focused on full-time academic staff from four leading universities located in South-Western Nigeria. These universities were all government-accredited and were selected based on criteria such as age, funding levels, and rankings. They demonstrated exceptional performance, not only being top-rated in the region but also at the national and international levels [120]. The constructs were measured at the individual level. Participants in the study were assured that their information would remain completely confidential and be utilized solely for academic research purposes.

Prior to commencing data analysis, this study determined the minimum sample size. This research identified the appropriate sample size by utilizing the Krejcie and Morgan [121] table for determining sample sizes [122]. In partial least squares based structural equation modelling (PLS-SEM), Karaboga, Erdal [25] proposed that a sample size between 100 and 200 is adequate. Additionally, Hair, Black [123] suggested that when conducting research with Structural Equation Modelling, the sample size should be no less than 100. Thus, the sample size of this study is 314 academic staffs working Nigerian federal and state universities, which is adequate for evaluating proposed relationships. In line with suggestions from well-known literature [124,125], this study used convenience sampling. Academics, in particular, face significant time constraints due to the demanding nature of their roles, which encompass research, teaching, and community service duties. Consequently, the convenience sampling technique provided both accessibility and adaptability [126].

Data were collected through Google Form questionnaire that was distributed to academics at the university via email. An email was sent to academics outlining the study’s purpose, and those interested in participating in the survey are expected to complete it. Participation in the survey was optional, and the study received approval from the researchers’ Ethics Committee. Informed consent was obtained in written form from each participant prior to their involvement in the study. The questionnaire was distributed during the period from June 2023 to September 2023. This specific time frame was chosen intentionally, as it coincided with the end of the semester when participants were likely to have more available time to complete the survey. A total of 300 emails were distributed, resulting in 249 responses being collected. Out of the total 249 responses, 63% (156) were from males and 37% (93) from females. The survey achieved a 79% response rate. A majority of participants (85%) held doctoral degrees, while the remaining 15% possessed master’s degrees. The academic positions of respondents varied, with 13% being Lecturers, 65% serving as Assistant Professors, and 22% holding positions as Associate or Full Professors.

For the statistical analysis in this research, data coding and assumption verification were conducted using SPSS version 26. Structural Equation Modeling (SEM) was employed to assess latent variables and evaluate construct validity [127]. Structural equation modeling (SEM) is a technique used in multivariate statistical analysis that allows researchers to examine the structural relationships between observed variables and latent constructs [128], determining the relative influence of each variable on the study’s proposed model. To evaluate the conceptual model shown in Fig.1, SmartPLS analysis was conducted. This study employs PLS analysis because it is a component-based method that does not impose stringent requirements on sample size or residual distribution [129]. Additionally, PLS analysis allows for the concurrent assessment of both a theoretical structural model and a measurement model [130]. In past research, PLS-SEM has been employed to analyze data related to employee creativity and innovative work behavior, making it a suitable choice for exploratory studies that integrate explanatory and predictive approaches [25,42]. SmartPLS 4.0 software is utilized to analyze measurement model, ensuring they possess the correct psychometric properties, and to test the research model and hypotheses, particularly focusing on the structural model.

### 3.2 Scales

This survey aims to explore how university staff members perceive their capacity for creativity and innovation in their work environment. This research employed a questionnaire for data gathering, which included four variables: HPWS, employee creativity, innovative work behaviour, and knowledge sharing. This research employed scales that have been utilized in numerous prior studies, as all these variables have been previously identified and measured (Refer to S1 File Appendix A in supporting information, Table A1. Research instruments). By doing so, the study made use of instruments that have already demonstrated reliability and validity. However, the questionnaires were adapted to align with the specific scope and objectives of this study. The questions were based on a five-point Likert scale, offering respondents five levels of agreement. The Likert scale is a common tool in the Social Sciences [17]. The scales were as follow:

#### 3.2.1 Innovative work behaviour (IWB).

Innovative behaviour involves effectively putting creative ideas into practice within a company [131]. Innovation involves both creating and applying new ideas, which demands a diverse set of behaviours from individuals [132,133]. While some people may display the full range of innovative behaviours, others may only demonstrate one or a few of these actions [132,133]. Innovative work behaviour was measured using a 6-item scale [132,133]. Responses were made on a five-point Likert-type scale ranging from 1 = “strongly disagree” to 5 = “strongly agree.” Cronbach’s alpha on this scale was 0.89. A sample item is “I often promote and champion ideas to others”.

#### 3.2.2 Employee creativity (EMP-CRE).

Creativity is defined as the generation of original and practical ideas by an individual or a small group collaborating [131]. In this study, employee creativity was measured using a 13‑item scale developed by Nasifoglu Elidemir, Ozturen [2], originally designed to assess creativity from the manager’s perspective. To fit the study’s focus, all items were rephrased from the third-person (e.g., “He/She suggests new ways to achieve goals and objectives”) to the first-person (e.g., “I suggest new ways to achieve goals and objectives”), thereby capturing self-assessed creativity directly from employees. This adaptation ensured alignment with the research objective of evaluating creativity from the employees’ viewpoint rather than through managerial evaluation. Self-reporting enabled respondents to provide richer, context-specific insights into how they perceive and demonstrate creativity in their roles. To ensure validity, content expert reviews and pilot testing were conducted to assess item clarity and relevance, followed by statistical evaluation of reliability and construct validity. The modification retained the original meaning and construct validity of the scale while making it appropriate for a self-report format. All items were rated on a five-point Likert scale ranging from 1 (strongly disagree) to 5 (strongly agree).

#### 3.2.3 High performance work system (HPWS).

HPWS was evaluated using a 12-item scale, which was modified from Wang, Zhu [134] for the purposes of the present research. for this study. Participants were asked to rate how much their organization provides these practices. The following are some examples: “To improve interpersonal skills, the company will provide training opportunities” and “The company encourages me to work hard”. The Cronbach’s α for this scale was 0.88, reflecting an acceptable level of measurement reliability.

#### 3.2.4 Knowledge sharing.

In this research, knowledge sharing was assessed using four items from the scale created by Xu and Suntrayuth [135]. An example item is “In my daily work, I take the initiative to impart academic knowledge to colleagues.” The Cronbach’s α for this instrument was 0.895, indicating that the measurement reliability is acceptable.

## 4. Results and findings

The study employed Structural Equation Modeling (SEM), which consists of measurement and structural models. The measurement model validates the measurements through confirmatory factor analysis (CFA), while the structural model examines the relationships between variables [136]. Prior to implementing SEM, various assumptions had to be verified to ensure the data’s reliability for subsequent statistical analyses.

### 4.1 Measurement model

**Fig 2:**
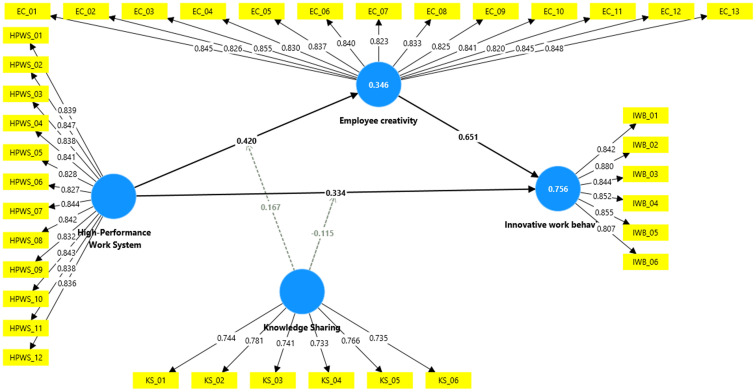
Measurement model.

In line with the recommendations by Hair, Hollingsworth [137], the evaluation of the measurement model involved conducting factor analysis, assessing reliability and internal consistency, and examining both discriminant and convergent validity. Fig 2 presents the measurement model used in this study. This research employed Cronbach’s alpha (CA) and composite reliability (CR) to assess reliability. Table 1 displays the CA and CR values for employee creativity (0.964, 0.968), high performance work system (0.961, 0.966), innovative work behaviour (0.921, 0.938), and knowledge sharing (0.845, 0.885) respectively. According to Hair et al. (2011), CA and CR values exceeding 0.70 are deemed satisfactory. The findings of this study demonstrate that all values fall within an acceptable range. Moreover, a possible multicollinearity between HPWS, employee creativity, knowledge sharing and innovative work behaviour detected using the variance inflation factor (VIF). Yesuf, Getahun [138] suggest that the Variance Inflation Factor (VIF) should remain below 5. The observed VIF values ranged from 1.000 to 3.114, falling within the acceptable limits. This indicates that the dataset was not affected by multicollinearity problems.

**Table 1:** Statistical reliability.

Construct	Items	Loadings	Alpha	CR	AVE	VIF
Employee Creativity	EC_01	0.845	0.964	0.964	0.699	3.100
	EC_02	0.826				2.800
	EC_03	0.855				3.203
	EC_04	0.830				2.856
	EC_05	0.837				2.950
	EC_06	0.840				3.004
	EC_07	0.823				2.712
	EC_08	0.833				2.923
	EC_09	0.825				2.795
	EC_10	0.841				3.041
	EC_11	0.820				2.756
	EC_12	0.845				3.103
	EC_13	0.848				3.114
HPWS	HPWS_01	0.839	0.921	0.922	0.702	2.955
	HPWS_02	0.847				3.051
	HPWS_03	0.838				3.035
	HPWS_04	0.841				2.926
	HPWS_05	0.828				2.790
	HPWS_06	0.827				2.920
	HPWS_07	0.844				2.987
	HPWS_08	0.842				2.977
	HPWS_09	0.832				2.944
	HPWS_10	0.843				2.967
	HPWS_11	0.838				2.890
	HPWS_12	0.836				2.909
Knowledge sharing	KS_01	0.744	0.845	0.848	0.563	2.491
	KS_02	0.781				3.029
	KS_03	0.741				2.449
	KS_04	0.733				2.582
	KS_05	0.766				2.673
	KS_06	0.735				2.111
Innovative work behaviour	InnWB_01	0.842	0.921	0.922	0.717	1.584
	InnWB_02	0.880				1.794
	InnWB_03	0.844				1.677
	InnWB_04	0.852				1.601
	InnWB_05	0.855				1.663
	InnWB_06	0.807				1.661

The study demonstrated both convergent and discriminant validity. Convergent validity was confirmed through Average Variance Extracted (AVE) values, which surpassed the 0.50 threshold recommended by [137]. Specifically, the AVE values exceeded this limit for HPWS (0.702), employee creativity (0.699), knowledge sharing (0.563), and innovative work behaviour (0.717), thus validating convergent validity. Further support for this validity was evident in the acceptable loading levels of all questionnaire items, ranging from 0.563 to 0.717 (as shown in Table 1). Additionally, discriminant validity was verified using two methods: The Fornell–Larcker criterion and the Heterotrait–Monotrait ratio of correlations, aligning with the guidelines proposed by Fornell and Larcker [139] and Henseler, Ringle [140] These results are presented in Tables 2 and 3. As demonstrated in Table 2, Fornell and Larcker’s test results exceed the correlations between variables. Additionally, the HTMT values fell below the 0.90 threshold, in accordance with the criterion established by Henseler, Ringle [140] (refer to Table 3 for these values). These findings substantiate the discriminant validity of the present study.

**Table 2:** Discriminant validity (Fornell– Larcker).

	Employee creativity	HPWS	Innovative work behaviour	Knowledge sharing
Employee creativity	0.836			
HPWS	0.455	0.838		
Innovative work behaviour	0.803	0.645	0.847	
Knowledge Sharing	0.400	0.147	0.342	0.750

**Table 3:** Discriminant validity (Heterotrait– Monotrait).

	Employee creativity	HPWS	Innovative work behaviour	Knowledge sharing
Employee creativity				
HPWS	0.471			
Innovative work behaviour	0.851	0.684		
Knowledge Sharing	0.440	0.162	0.385	

### 4.2 Structural model

**Fig 3:**
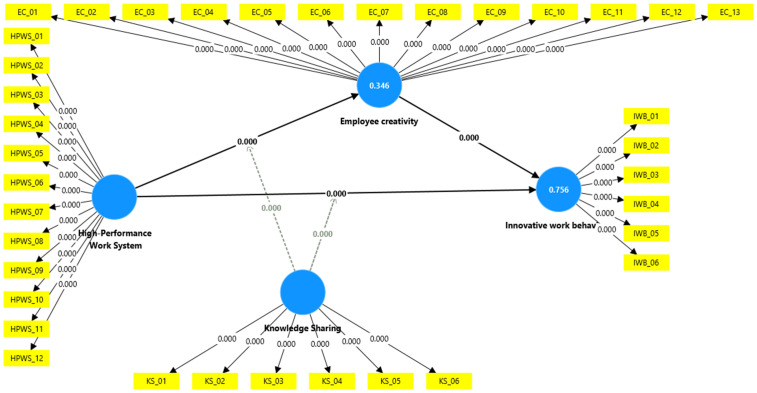
Structural model.

After confirming the measurement model, the structural model was assessed to examine the underlying relationships (see Fig 3). According to the suggestions of Henseler, Ringle [140] and Hair, Hollingsworth [137], this assessment involves analysing the obtained coefficient of determination (R2), path coefficients (β), and their significance using (T) statistics, along with the Stone–Geisser (Q2) criterion, to evaluate the predictive relevance of the inner model. Endogenous latent variables with coefficients of determination (R^2^) values of 0.756 and 0.346 exhibit substantial and weak explanatory power, respectively [123]. Table 4 shows that R^2^ (Creativity) = 0.346 and R^2^ (IWB) = 0.756, the structural model for innovative work behaviour had satisfactory in-sample predictive power. The coefficient of determination (R2) for creativity was 0.346, signifying that employee creativity explained 34.6% of the variance in innovative work behaviour. This represents a moderate level of variance. The Stone–Geisser value (Q2) was above the zero threshold, suggesting that employee creativity has predictive power over innovative work behaviour. Therefore, the findings of this study were significant, and the predictive relevance of the study model was confirmed [141].

**Table 4:** Saturated model results.

	R-square	R-square adjusted	Q^2^ predict	SPMR
Employee creativity	0.346	0.341	0.332	0.034
Innovative work behaviour	0.756	0.753	0.471	

Standardized root mean square residual (SRMR); determination of coefficient (R2); cross-validiated redundancy (Q2).

According to the PLS–SEM findings, (H1) testing the direct effects of high performance work system on innovative work behaviour revealed a significant relationship (β = 0.334, t = 9.597, p = 0.000). H2 found a significant relationship between high performance work system and employee creativity (β = 0.420, t = 12.401, p = 0.000). Hence, H2 was supported. The results of the study indicated that the relationship between employee creativity and innovative work behaviour was significant (β = 0.651, t = 24.346, p = 0.000). Thus, H3 was supported (see Table 5).

**Table 5:** Results of hypothesis testing (direct effect).

	Beta	STDEV	t-value	P values	Decision
Employee creativity - > Innovative work behaviour	0.651	0.027	24.346	0.000	Supported
High-Performance_ Work System - > Employee creativity	0.420	0.034	12.401	0.000	Supported
High-Performance_ Work System - > Innovative work behaviour	0.334	0.035	9.597	0.000	Supported

Hypothesis H4 proposes that employee creativity mediates the relationship between HPWS and innovative work behaviour (IWB). Applying Baron and Kenny [142]’s mediation procedure, three conditions were met: (1) HPWS significantly predicts IWB, as demonstrated in H1; (2) HPWS significantly affects employee creativity, which in turn also significantly predicts IWB; and (3) the indirect effect of HPWS on employee creativity through creative resources is statistically significant (β = 0.274, t = 10.007, p = 0.000). These results therefore provide partial support for H4. The findings revealed that employee creativity partially mediates this relationship, as both direct and indirect effects were significant, thus confirming hypothesis H4. Notably, previous research has often overlooked or considered employee creativity solely as a final outcome of HPWS. The results of this study indicate that employee creativity facilitates innovative work behaviour among academic staff members.

**Fig 4:**
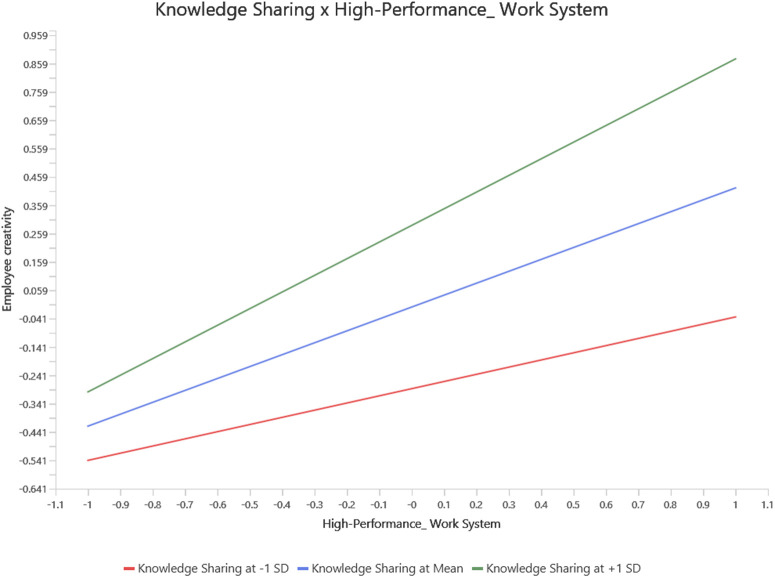
Simple slop analysis for knowldeg sharing moderation model (HPWS and employee creativity).

**Fig 5:**
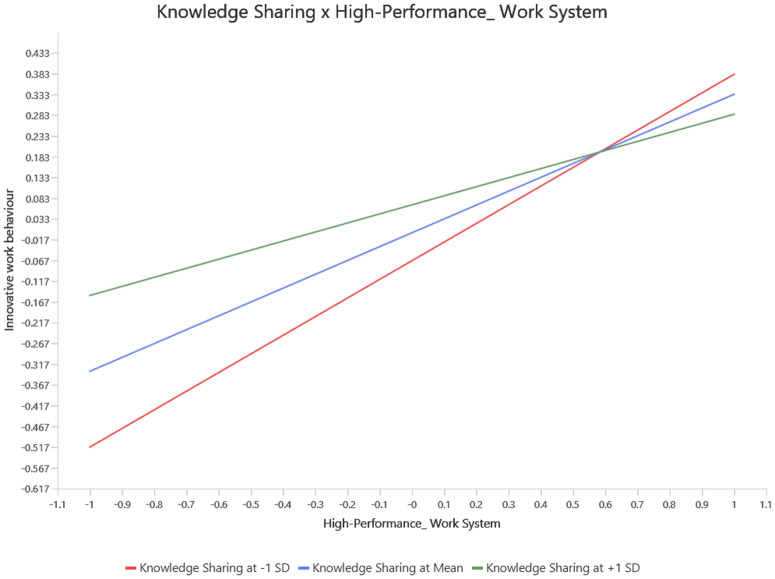
Simple slop analysis for knowldeg sharing moderation model (HPWS and inovative work behaviour).

Lastly, this research also examined the moderating effect of knowledge sharing on the relationships between HPWS and employee creativity, as well as HPWS and innovative work behaviour (see Table 6). Previous studies have not explored this moderating mechanism [108]. The influence of HPWS on innovative work behaviour and employee creativity is altered when the moderating variable (knowledge sharing) increases by one standard deviation [143]. The significant beta coefficient (β = 0.109, t = 4.716, p = 0.000) for the interaction terms of HPWS, employee creativity, and innovative work behaviour supports the hypothesis that knowledge sharing affects the impact of HPWS on IWB and employee creativity. Consequently, when knowledge sharing is high, the positive effect of HPWS on IWB and employee creativity is stronger. Therefore, both H5 and H6 were confirmed. SmartPLS was utilized to conduct a simple slope analysis, which illustrated the moderating effect as shown in Figs 4 and 5.

**Table 6:** Results of hypotheses testing (mediating moderating effect).

	Beta	STDEV	T value	P values	Decision
High performance work system - > Employee creativity - > Innovative work behaviour	0.274	0.027	10.007	0.000	Supported
Knowledge Sharing x High-Performance_ Work System - > Employee creativity - > Innovative work behaviour	0.109	0.023	4.716	0.000	Supported

The analysis revealed that when knowledge sharing is elevated, HPWS has a more pronounced positive impact on innovative work behaviour (IWB) and employee creativity. This suggests that under conditions of high knowledge sharing, employees exhibit greater levels of innovation and creativity. Conversely, when knowledge sharing is low, the positive effect of HPWS on these outcomes is weaker, indicating that limited knowledge exchange may constrain the ability of HPWS to enhance employee innovative behaviours and creative output. In essence, the simple slope analysis helps clarify that knowledge sharing plays a vital moderating role: it enhances the effectiveness of HPWS in promoting employee innovation and creativity. This highlights the importance for organizations to foster a culture of knowledge sharing to fully leverage the potential of HPWS in driving innovative and creative performance among employees.

## 5. Discussion

This research proposed a moderated mediation model to elucidate the relationship between HPWS and innovative work behaviour. The framework was examined at the individual level, focusing on academic staffs’ experience of high performance work systems on IWB, as this approach provides more meaningful insights into outcomes at the institutional level. The research utilizes social exchange theory to suggest that employees’ perceptions of HPWS impact their innovative actions both directly and indirectly through the mediation of employee creativity and the moderation of knowledge-sharing behaviour. The research focused on Nigerian higher education institutions. The relationship between high performance work systems (HPWS) and innovative work behaviour has been examined in various empirical studies. Whilst some research has demonstrated a significant correlation [32,144–146], a study conducted by Abdillah and Asfar [147] found no significant link between HPWS and innovative work behaviour among elementary school teachers in Pekaian District. The inconsistent findings, including the non-significant direct relationship, may be explained by the influence of additional variables on the association between HPWS and innovative work behaviour.

This research underscores the significance of a high-performance work system (HPWS) in fostering innovative work behaviour (IWB). Drawing on social exchange theory’s reciprocity norms, the study posited that HPWS would encourage employees’ IWB, a hypothesis subsequently confirmed by the findings. The positive impact of HPWS on IWB stems from the favourable signals it sends to employees regarding their value to the organisation. These signals may be conveyed through the AMO (ability, motivation, and opportunities) theory by enhancing employees’ capabilities (via training and development), encouraging them to exceed their job requirements (through involvement in decision-making), and providing opportunities for growth and job security [145]. However, employees may reciprocate with extra-role behaviour and increased innovation, aligning with the principles of social exchange theory. This evaluation was significant because the investigation of this relationship is primarily carried out in settings outside of education [17]. Therefore, this research aimed to explore this significant relationship within the educational setting in Nigeria by employing a cross-sectional method.

The results of this research support the claim that a set of strategic HRM practices can have a positive impact on employee behaviour [32,34]. According to Yuan and Xie [24], the implementation of a high-performance work system is critical for fostering the creation of innovative and significant concepts. Research by Tang, Yu [41] highlights that development-focused HR practices, as part of HPWS, have a beneficial impact on employee creativity. Van De Voorde, Paauwe [148] observed that combining HRM practices into a cohesive HPWS package produces a synergistic outcome, leading to enhanced employee functionality and performance in the work environment. However, this perspective was challenged by Kroon, Van de Voorde [149], who contended that implementing HPWS could potentially be detrimental to employee well-being, as these systems might foster exploitation. This study’s results, however, refuted this criticism, revealing that the application of HPWS is advantageous, as it positively influences employee creativity and innovative behaviour.

Employee creativity and innovative behavior are essential drivers of organizational success, especially within public sector institutions [150]. The employee idea journey begins with creative behavior (idea exploration and generation) and progresses to innovative behavior (idea championing and implementation) [151]. This research demonstrated a significant and positive correlation between employee creativity and innovative work behaviour (IWB) aligning with the outcomes reported by Al-Ajlouni [17]. While considerable attention has been given to studying employee creativity in corporate settings, there is a notable lack of research examining academic staff creativity within university environments [43,58]. A creative faculty member possesses the ability to find better solutions to challenges across various aspects of life, including their work. Rather than approaching issues through conventional logical means, they can employ innovative methods and examine situations from diverse perspectives, thereby enhancing the quality and effectiveness of the educational process.

This research contributes to the existing body of knowledge on employee creativity by demonstrating that the adoption of High-Performance Work Systems (HPWS) fosters creative thinking among staff members, a finding that stands in contrast to the conclusions drawn by Al-Ajlouni [17]. Previous studies has investigated the link between high-performance work systems (HPWS) and employee creativity as an end result [34,41]. Nevertheless, Riana, Wiagustini [34] suggest that creativity serves as the initial stage of creative behaviour, generating innovative ideas that could eventually lead to innovation. Partially inspired by this invitation to address a gap in the existing literature, this investigation proposes and evaluates a hypothesis concerning the impact of HPWS on innovative work behaviour through employee creativity. The results broaden previous studies on the association between HPWS and innovative work behaviour. Furthermore, our observation that employees perceive HPWS as a demonstration of creative behaviour and respond with innovative behaviour offers additional empirical support for social-exchange theory.

To gain in-depth insights into the relationship between high-performance work systems (HPWS), employee creativity and innovative work behaviour, this study explored the positive moderating role of knowledge sharing. According to Riana, Wiagustini [34], employee creativity and innovative work behaviour can be formed through a knowledge sharing culture driven by university academic members. Academics play a vital role in generating knowledge, and enhancing the ways in which this knowledge is shared can lead to the advancement of high-quality education and boost institutional performance [152]. In an academic environment, knowledge is a valuable resource since all institutions are knowledge-based [102]. Although knowledge sharing is essential for academic success, there is a lack of research on knowledge sharing among faculty members in Nigerian higher education institutions [102]. This study investigated how knowledge sharing moderates the relationship between high-performance work systems (HPWS), employee creativity and innovative work behaviour.

Kim and Lee [153] highlighted the importance of individuals sharing their knowledge to foster innovative behavior. Existing research has recognised knowledge sharing as a key influencing employee creativity and innovative behaviour in the workplace [45,54]. The research results indicated that knowledge sharing acted as a moderator in the relationship between HPWS, employee creativity, and innovative behaviour. This finding contrasts with the work of Faris Hussain, Hanifah [108] who discovered that knowledge sharing did not moderate the link between management support and innovative work behaviour. However, the results of this study demonstrated that positive relationship between HPWS, employee creativity and innovative work behaviour is stronger when knowledge sharing is high. It was also suggested that management should motivate employees to exchange their knowledge to foster innovative behavior [154,155]. Although researchers have highlighted the significance of knowledge sharing in promoting innovative behaviour, Rai, Ghosh [156] and Faris Hussain, Hanifah [108] underlined the restriction of study on the relationship between knowledge sharing and IWB, especially as a moderator. Additional studies are necessary to investigate how knowledge sharing moderates the relationship between creativity and innovative behaviour, particularly within higher education institutions, with a specific focus on Nigeria.

## 6. Implications

This study examined the relationship between high-performance work systems (HPWS) and innovative work behaviour among academic staff in Nigeria. It also explored the mediating effect of employee creativity between HPWS and IWB, while having knowledge sharing as a moderator. This research addresses the concern raised by Al-Ajlouni [17] regarding the slow progress in establishing a definitive causal connection between HRM and innovation, particularly in understanding the underlying mechanisms. To advance this field, the study proposes an integrated mediated moderated framework that examines the relationship at the individual level. By focusing on employee self-assessments of their behaviours and attitudes, the research makes a significant contribution, as it yielded results that support the proposed model. This approach differs from previous studies, which have primarily concentrated on macro-level outcomes such as organizational innovation [17]. Moreover, the research contributes to the existing body of knowledge on human resource management (HRM) and higher education institutions. Although scholars and researchers in commercial enterprises widely recognize the role of HPWS in promoting innovation and creativity, there is a scarcity of studies examining how HPWS affects creativity and innovative work behaviour in academic settings. This research sought to fill these gaps in the existing literature.

This study’s findings revealed that academic members perceive HPWS as a catalyst for creativity and reciprocate this with IWB; this extends the empirical evidence for social exchange theory. Academics provide universities with essential insights into human resource policies and systems, offering innovative approaches, facilitators, interventions, and recommendations that support the promotion of innovative work behavior (IWB) [4]. This research also indicates that knowledge sharing as a moderator can strengthen the relationship between HPWS, creativity and innovative work behaviour. As creativity and innovation stem from employees’ minds and hearts, it is essential for managers to foster an environment that encourages idea sharing and experimentation. This supportive atmosphere should allow employees to feel comfortable sharing their ideas and experimenting with them, even if it means risking failure [48]. This study provides valuable information to higher education system on the relevance of HPWS, knowledge sharing, creativity and innovation.

Most research on HPWS and innovative work behaviour concentrates on corporate environments, leading to limited understanding of how these dynamics manifest in academic settings, particularly among faculty members. The distinguishing features of academia—such as the dual emphasis on research and teaching, different incentive structures, and more collegial governance models—can significantly influence how HPWS impact innovative behaviours compared to more hierarchical corporate contexts. Additionally, faculty members often operate with different types and levels of autonomy and face distinct motivational challenges. Therefore, these differences suggest that the mechanisms linking HPWS to innovation may vary between educational and corporate sectors, underscoring the need for focused studies within the academic domain.

The moderation of the relationship between High-Performance Work System (HPWS) and employee outcomes by knowledge sharing offers significant theoretical insights through the integration of the AMO and Social Exchange Theory (SET) frameworks. The AMO model proposes a motivational pathway, arguing that HR practices like HPWS promote growth, learning, and development by addressing both personal and organizational needs, thereby enhancing motivation, providing opportunities, and developing employee abilities [9]. While HPWS mainly focuses on improving employees’ abilities and motivation through initiatives such as training, incentives, and support, the opportunity component is equally important. This component includes elements like knowledge sharing, job autonomy, and participation in decision-making, which provide employees with the necessary environment and resources to effectively apply their skills and motivation. Without these opportunities, enhancements in ability and motivation alone may not lead to significant increases in creativity and innovation. Therefore, this underscores the importance of promoting opportunity-driven behaviors such as knowledge sharing to fully harness the benefits of HPWS. From the SET perspective, HPWS reflects organizational investments that are reciprocated by employee contributions, but the success of this exchange depends heavily on social interactions and the quality of reciprocal relationships, which are facilitated by knowledge sharing. When knowledge sharing is high, it fosters trust and builds social capital, strengthening the reciprocal exchange process and driving greater employee creativity and innovative behaviors.

## 7. Limitations

The present study is subject to several limitations. Primarily, it examined knowledge sharing as the sole moderator between HPWS, employee creativity, and innovative work behaviour, thus limiting consideration of other potential contextual moderators such as organizational climate, leadership style, and team dynamics. Future research should explore these additional moderators and also consider the possible mediating or moderating role of innovation propensity to provide greater insight into the mechanisms linking HPWS to employee outcomes.

This study employed a convenience sampling approach, which, by its nature, limits the generalizability and external validity of the findings. Because participants were not selected through a random or probability-based method, the sample may not fully represent the broader population, and results should be interpreted with caution. Future studies would benefit from adopting probability-based sampling methods to improve representativeness and external validity. Moreover, while this study utilized a quantitative approach consistent with most empirical work in the field, incorporating mixed methods could have facilitated a more comprehensive and nuanced investigation. Finally, the cross-sectional design captured attitudes and behaviours only at a single point in time, which limits the ability to infer causality. While the theoretical model proposes causal directions, the nature of the data prevents definitive causal conclusions. Longitudinal research would better capture the dynamic nature of these constructs and provide a stronger basis for confirming the causal relationships proposed in this study.

## 8. Conclusion

This study explored the relationships among high performance work systems, knowledge sharing, employee creativity, and innovative work behaviour within Nigerian higher education institutions. The findings consistently support the proposed conceptual framework: HPWS positively influence innovative work behaviour among academic staff, and both knowledge sharing and employee creativity serve as important mechanisms in this process. Specifically, knowledge sharing not only enhances the direct effect of HPWS on innovative work behaviour, but also amplifies employee creativity, which in turn mediates the relationship between HPWS and innovation. These results underscore the importance of collaboration and creativity in turning high-performance work systems into tangible innovation. For leaders and policymakers in Nigerian higher education, creating environments that support knowledge sharing and creative thinking can greatly enhance the effectiveness of HPWS, leading to increased innovation and improved institutional performance. Together, these insights provide actionable guidance for enhancing institutional effectiveness and promoting sustainable, innovative growth.

## Supporting information

S1 File Appendix A. Table A1. Research instruments. (DOCX)

## References

[1] Elrehail H, Emeagwali OL, Alsaad A, Alzghoul A. The impact of transformational and authentic leadership on innovation in higher education: the contingent role of knowledge sharing. Telematics and Informatics. 2018;35(1):55–67. doi: 10.1016/j.tele.2017.09.018

[2] Nasifoglu Elidemir S, Ozturen A, Bayighomog SW. Innovative behaviors, employee creativity, and sustainable competitive advantage: a moderated mediation. Sustainability. 2020;12(8):3295. doi: 10.3390/su12083295

[3] Tri HT, Nga VT, Sipko J. Predicting overall staffs’ creativity and innovative work behavior in banking. Manag Market Challeng Knowled Soc. 2019;14(2):188–202. doi: 10.2478/mmcks-2019-0013

[4] AlEssa HS, Durugbo CM. Systematic review of innovative work behavior concepts and contributions. Manag Rev Q. 2021;72(4):1171–208. doi: 10.1007/s11301-021-00224-x

[5] Namono R, Kemboi A, Chepkwony J. Enhancing innovative work behaviour in higher institutions of learning: the role of hope. World J Entrepreneurship Manag Sustain Devlop. 2021;ahead-of-print(ahead-of-print). doi: 10.1108/wjemsd-07-2020-0073

[6] Ahmad T. Universities preparing students for future challenges of family business enterprises. WJEMSD. 2020;16(2):57–69. doi: 10.1108/wjemsd-05-2020-111

[7] Dai Y, Qin S, Tang YM, Hou J. Fostering employees’ innovative behavior: the importance of proactive personality and work-related flow. Acta Psychol (Amst). 2024;246:104278. doi: 10.1016/j.actpsy.2024.104278 38670040

[8] Ogunode NJ, Musa A. Higher education in Nigeria: challenges and the ways forward. Elect Res J Behav Sci. 2020;3.

[9] Odiaka KU. Employee engagement and its importance to HPWPs (High Performance Work Practices), employee outcome relationship in the Nigerian hotel sector. United Kingdom: University of Salford; 2020.

[10] Orth M, Volmer J. Daily within-person effects of job autonomy and work engagement on innovative behaviour: the cross-level moderating role of creative self-efficacy. Euro J Work Organ Psychol. 2017;26(4):601–12. doi: 10.1080/1359432x.2017.1332042

[11] Saether EA. Motivational antecedents to high-tech R&D employees’ innovative work behavior: self-determined motivation, person-organization fit, organization support of creativity, and pay justice. J High Tech Manag Res. 2019;30(2):100350. doi: 10.1016/j.hitech.2019.100350

[12] Mutonyi BR, Slåtten T, Lien G. Empowering leadership, work group cohesiveness, individual learning orientation and individual innovative behaviour in the public sector: empirical evidence from Norway. Int J Public Leadership. 2020;16(2):175–97. doi: 10.1108/ijpl-07-2019-0045

[13] Bawuro FA, Shamsuddin A, Wahab E, Chidozie CC. Prosocial motivation and innovative behaviour: an empirical analysis of selected public university lecturers in Nigeria. Inter J Scient Technol Res. 2019;8(9):1187–94.

[14] Khan MM, Mubarik MS, Islam T. Leading the innovation: role of trust and job crafting as sequential mediators relating servant leadership and innovative work behavior. Euro J Inniv Manag. 2020;24(5):1547–68. doi: 10.1108/ejim-05-2020-0187

[15] Pradana ER, Suhariadi F. The effect of job crafting on innovative behavior through mediation work engagement. Airlangga J Innov Manang. 2020;1(1):77. doi: 10.20473/ajim.v1i1.19402

[16] Sharma A, Nambudiri R. Work engagement, job crafting and innovativeness in the Indian IT industry. Personnel Review. 2020;49(7):1381–97. doi: 10.1108/pr-11-2019-0607

[17] Al-Ajlouni MI. Can high-performance work systems (HPWS) promote organisational innovation? Employee perspective-taking, engagement and creativity in a moderated mediation model. Int J. 2020;43(2):373–97. doi: 10.1108/er-09-2019-0369

[18] Wernerfelt B. A resource‐based view of the firm. Strat Manag J. 1984;5(2):171–80. doi: 10.1002/smj.4250050207

[19] Dara D. An investigation of faculty members’ job autonomy, work satisfaction, and innovative work behavior indicators. Int J Acad Res Business Soc Sci. 2023;13(12). doi: 10.6007/ijarbss/v13-i12/19852

[20] Woodman RW, Sawyer JE, Griffin RW. Toward a theory of organizational creativity. Acad Manag Rev. 1993;18(2):293. doi: 10.2307/258761

[21] Amabile TM, Conti R, Coon H, Lazenby J, Herron M. Assessing the work environment for creativity. Acad Manag J. 1996;39(5):1154–84. doi: 10.2307/256995

[22] Amabile TM, Pratt MG. The dynamic componential model of creativity and innovation in organizations: making progress, making meaning. Res Organ Behav. 2016;36:157–83. doi: 10.1016/j.riob.2016.10.001

[23] O’Regan N, Ghobadian A, Gallear D. In search of the drivers of high growth in manufacturing SMEs. Technovation. 2006;26(1):30–41. doi: 10.1016/j.technovation.2005.05.004

[24] Yuan X, Xie L. High-performance work system and employee creativity. In: International Conference on Creative Industry and Knowledge Economy (CIKE 2022). Atlantis Press; 2022.

[25] Karaboga T, Erdal N, Karaboga HA, Tatoglu E. Creativity as a mediator between personal accomplishment and task performance: a multigroup analysis based on gender during the COVID-19 pandemic. Curr Psychol. 2022;:1–13. doi: 10.1007/s12144-021-02510-z 35002188 PMC8727074

[26] Yulianti P. Membangun perilaku inovatif dosen perguruan tinggi. Jurnal Studi Manajemen Dan Bisnis. 2016;3(1):31–9.

[27] Kim B-J, Nurunnabi M, Kim T-H, Jung S-Y. Does a Good Firm Breed Good Organizational Citizens? The Moderating Role of Perspective Taking. Int J Environ Res Public Health. 2019;16(1):161. doi: 10.3390/ijerph16010161 30626143 PMC6338899

[28] Mbuni A. The impact of meaningful work on innovative work behavior mediated through employee engagement. Capella University; 2021.

[29] Phairat P, Potipiroon W. High performance work systems and innovative work behavior among telecom employees: the roles of organizational climate for innovation and psychological empowerment. ABAC Journal. 2022;42(3):214–31. doi: 10.14456/abacj.2022.30

[30] Shin SJ, Jeong I, Bae J. Do high-involvement HRM practices matter for worker creativity? a cross-level approach. Inter J Human Res Manag. 2016;29(2):260–85. doi: 10.1080/09585192.2015.1137612

[31] Fragoso P, Chambel MJ, Castanheira F. High-performance work systems (HPWS) and individual performance: the mediating role of commitment. Mil Psychol. 2021;34(4):469–83. doi: 10.1080/08995605.2021.2010429 38536383 PMC10013567

[32] Miao R, Lu L, Cao Y, Du Q. The high-performance work system, employee voice, and innovative behavior: the moderating role of psychological safety. Int J Environ Res Public Health. 2020;17(4):1150. doi: 10.3390/ijerph17041150 32059559 PMC7068291

[33] Miao R, Cao Y. High-performance work system, work well-being, and employee creativity: cross-level moderating role of transformational leadership. Int J Environ Res Public Health. 2019;16(9):1640. doi: 10.3390/ijerph16091640 31083469 PMC6539597

[34] Riana IG, Wiagustini NLP, Aristana IN, Rihayana IG, Abbas EW. HIgh-performance work system in moderating entrepreneurial leadership, employee creativity and knowledge sharing. Polish J Manang Stud. 2020;21(1):328–41. doi: 10.17512/pjms.2020.21.1.24

[35] Benítez-Núñez C, Dorta-Afonso D, de Saá-Pérez P. High-performance work systems and employees’ outcomes in challenging contexts: the role of hindrance stressors. J Hospit Market Manag. 2024;33(6):807–30. doi: 10.1080/19368623.2024.2305638

[36] Cheng D, Huang J, Wang X, Zhao S. Structure of high-performance work system and new product entry strategy: configuration based on manufacturing enterprises in China. Chinese Manag Stud. 2025;19(5):1658–80. doi: 10.1108/cms-11-2023-0614

[37] Li C, Naz S, Khan MAS, Kusi B, Murad M. An empirical investigation on the relationship between a high-performance work system and employee performance: measuring a mediation model through partial least squares-structural equation modeling. Psychol Res Behav Manag. 2019;12:397–416. doi: 10.2147/PRBM.S195533 31239795 PMC6551595

[38] Mao N, Song H, Han Y. High-performance work systems and influence processes on employees’ attitudes. Inter J Manpower. 2013;34(7):736–52. doi: 10.1108/ijm-07-2013-0157

[39] Jyoti J, Rani A. High performance work system and organisational performance: role of knowledge management. Personnel Rev. 2017;46(8):1770–95. doi: 10.1108/pr-10-2015-0262

[40] Ma Z, Long L, Zhang Y, Zhang J, Lam CK. Why do high-performance human resource practices matter for team creativity? The mediating role of collective efficacy and knowledge sharing. Asia Pac J Manag. 2017;34(3):565–86. doi: 10.1007/s10490-017-9508-1

[41] Tang G, Yu B, Cooke FL, Chen Y. High-performance work system and employee creativity. Personnel Rev. 2017;46(7):1318–34. doi: 10.1108/pr-09-2016-0235

[42] Escribá-Carda N, Balbastre-Benavent F, Teresa Canet-Giner M. Employees’ perceptions of high-performance work systems and innovative behaviour: the role of exploratory learning. Euro Manag J. 2017;35(2):273–81. doi: 10.1016/j.emj.2016.11.002

[43] Alsafadi Y, Al-Okaily M, Al-Okaily A, Shiyyab FS. The effects of high-performance work systems on the creativity of a faculty member: a moderated mediated analysis. Glob Knowled Memory Commun. 2024. doi: 10.1108/gkmc-03-2024-0158

[44] Hong L, Zainal SRM. The mediating role of organizational culture (OC) on the relationship between organizational citizenship behavior (OCB) and innovative work behavior (IWB) to employee performance (EP) in education sector of Malaysia. Global Busin Manag Res: An Inter J. 2022;14(3s).

[45] Battistelli A, Odoardi C, Vandenberghe C, Di Napoli G, Piccione L. Information sharing and innovative work behavior: the role of work‐based learning, challenging tasks, and organizational commitment. Hum Res Dev Quart. 2019;30(3):361–81. doi: 10.1002/hrdq.21344

[46] Nguyen TPL, Tran NM, Doan XH, Nguyen VH. The impact of knowledge sharing on innovative work behavior of Vietnam telecommunications enterprises employees. Manag Sci Lett. 2020;53–62. doi: 10.5267/j.msl.2019.8.016

[47] Janssen O. Job demands, perceptions of effort‐reward fairness and innovative work behaviour. J Occupat Organ Psyc. 2000;73(3):287–302. doi: 10.1348/096317900167038

[48] Swaroop P, Dixit V. Employee engagement, work autonomy and innovative work behaviour: An empirical study. Inter J Innov Creat Change. 2018;4(2):158–76.

[49] Bason C. Leading public sector innovation. Bristol: Policy Press; 2010.

[50] Hansen JA, Pihl-Thingvad S. Managing employee innovative behaviour through transformational and transactional leadership styles. Public Manag Rev. 2018;21(6):918–44. doi: 10.1080/14719037.2018.1544272

[51] De Spiegelaere S, Van Gyes G, Van Hootegem G. Innovative work behaviour and performance-related pay: rewarding the individual or the collective?. The International Journal of Human Resource Management. 2016;29(12):1900–19. doi: 10.1080/09585192.2016.1216873

[52] Namono R, Hojops OJP, Tanui S. Self-efficacy: implications for university employees’ innovativeness. Inter J Innov Sci. 2024;17(5):1235–52. doi: 10.1108/ijis-05-2023-0106

[53] Maulding MK. An examination of follower perceptions of leader behaviors influencing follower innovative work behavior. Indiana Wesleyan University; 2023.

[54] Asurakkody TA, Kim SH. Effects of knowledge sharing behavior on innovative work behavior among nursing students: mediating role of Self- leadership. Inter J Africa Nurs Sci. 2020;12:100190. doi: 10.1016/j.ijans.2020.100190

[55] Afsar B, Umrani WA. Transformational leadership and innovative work behavior. Europ J Innov Manag. 2019;23(3):402–28. doi: 10.1108/ejim-12-2018-0257

[56] Al-Hawari MA, Bani-Melhem S, Shamsudin FM. Determinants of frontline employee service innovative behavior. Manag Res Rev. 2019;42(9):1076–94. doi: 10.1108/mrr-07-2018-0266

[57] Alt D, Kapshuk Y, Dekel H. Promoting perceived creativity and innovative behavior: benefits of future problem-solving programs for higher education students. Think Skills Creat. 2023;47:101201. doi: 10.1016/j.tsc.2022.101201

[58] Saleem T, Rubbab DrU, Shahab DrH, Irshad DrM. High-performance work system and innovative work behavior: the mediating role of knowledge sharing and moderating role of inclusive leadership. Int J Bus Econ Aff. 2023;8(1). doi: 10.24088/ijbea-2023-81005

[59] Cao M, Zhao S, Chen J, Lv H. Employees’ HR attributions count: the effects of high-performance work systems on employees’ thriving at work and emotional exhaustion. Personnel Rev. 2023;53(4):835–56. doi: 10.1108/pr-09-2021-0632

[60] Ashiru J-A, Erdil GE, Oluwajana D. The linkage between high performance work systems on organizational performance, employee voice and employee innovation. J Org Change Manag. 2021;35(1):1–17. doi: 10.1108/jocm-02-2021-0039

[61] Hauff NV, Le Jeannic H, Lodahl P, Hughes S, Rotenberg N. Chiral quantum optics in broken-symmetry and topological photonic crystal waveguides. Phys Rev Res. 2022;4(2). doi: 10.1103/physrevresearch.4.023082

[62] Alsafadi Y, Altahat S. How ethical leadership and incivility tolerance affect intention to sabotage at Jordanian universities?. Orga Psychol. 2022;12(3):9–26. doi: 10.17323/2312-5942-2022-12-3-9-26

[63] Alsafadi Y, Altahat S. Human resource management practices and employee performance: the role of job satisfaction. J Asian Finan Econ Business. 2021;8(1):519–29. doi: 10.13106/jafeb.2021.vol8.no1.519

[64] Hu B, Hou Z, Mak MCK, Xu SL, Yang X, Hu T, et al. Work engagement, tenure, and external opportunities moderate perceived high-performance work systems and affective commitment. Soc Behav Pers. 2019;47(5):1–16. doi: 10.2224/sbp.7353

[65] Chiang Y-H, Hsu C-C, Shih H-A. Experienced high performance work system, extroversion personality, and creativity performance. Asia Pac J Manag. 2014;32(2):531–49. doi: 10.1007/s10490-014-9403-y

[66] Khawaldeh K, Alzghoul A. How knowledge sharing mediates the influence of high-performance work systems on employee intrepreneurial behavior: a moderation role of entrepreneurial leadership. Uncertain Supply Chain Manag. 2024;12(2):1365–78. doi: 10.5267/j.uscm.2023.11.002

[67] Lau C, Ngo H. The HR system, organizational culture, and product innovation. Inter Business Rev. 2004;13(6):685–703. doi: 10.1016/j.ibusrev.2004.08.001

[68] Jiang K, Lepak DP, Hu J, Baer JC. Does HRM facilitate employee creativity and organizational innovation? A study of Chinese firms. Whither Chinese HRM?. Routledge; 2012. 1264–94. doi: 10.5465/amj.2011.0088

[69] Chiang Y-H, Hsu C-C, Shih H-A. Experienced high performance work system, extroversion personality, and creativity performance. Asia Pac J Manag. 2014;32(2):531–49. doi: 10.1007/s10490-014-9403-y

[70] Rasheed MA, Shahzad K, Conroy C, Nadeem S, Siddique MU. Exploring the role of employee voice between high-performance work system and organizational innovation in small and medium enterprises. J Small Business Enterprise Develop. 2017;24(4):670–88. doi: 10.1108/jsbed-11-2016-0185

[71] Chan H-C, Chu K-M. Retracted: a multilevel perspective on high-performance work system, mindfulness, employee work well-being, and employee creative engagement. Sage Open. 2024;14(2). doi: 10.1177/21582440241242206

[72] Tran Huy P. How does high-performance work system influence employees’ creativity? The role of critical reflection and human resource management attribution. Int J Emerg Mark. 2023;20(2):638–59. doi: 10.1108/ijoem-03-2022-0508

[73] Blau PM. Justice in social exchange. Sociol Inq. 1964;34(2):193–206. doi: 10.1111/j.1475-682x.1964.tb00583.x

[74] He C, Gu J, Liu H. How do department high‐performance work systems affect creative performance? a cross‐level approach. Asia Pac J Human Res. 2017;56(3):402–26. doi: 10.1111/1744-7941.12156

[75] Dasgupta M. Driving creativity and innovation through emotional intelligence (EI): a systematic literature review. J Innov Manag. 2023;11(3):1–29. doi: 10.24840/2183-0606_011.003_0001

[76] Volery T, Tarabashkina L. The impact of organisational support, employee creativity and work centrality on innovative work behaviour. J Business Res. 2021;129:295–303. doi: 10.1016/j.jbusres.2021.02.049

[77] Walton AP. The impact of interpersonal factors on creativity. Inter J Entrepreneurial Behav Res. 2003;9(4):146–62. doi: 10.1108/13552550310485120

[78] AlMutairi Y. Assessing the strategic performance of the University of Ha’il by using balanced scorecard. J Edu/Al Mejlh Altrbwyh. 2024;38(151):121–54.

[79] Jiang K, Lepak DP, Hu J, Baer JC. How does human resource management influence organizational outcomes? A meta-analytic investigation of mediating mechanisms. Acad Manag J. 2012;55(6):1264–94. doi: 10.5465/amj.2011.0088

[80] Gong Y, Zhou J, Chang S. Core knowledge employee creativity and firm performance: the moderating role of riskiness orientation, firm size, and realized absorptive capacity. Personnel Psychol. 2013;66(2):443–82. doi: 10.1111/peps.12024

[81] Bauwens R, Audenaert M, Decramer A. Performance management systems, innovative work behavior and the role of transformational leadership: an experimental approach. J Org Effective Peop Perf. 2023;11(1):178–95. doi: 10.1108/joepp-03-2022-0066

[82] Ul Hassan SI, Din BH. The mediating effect of knowledge sharing among intrinsic motivation, high-performance work system and authentic leadership on university faculty members’ creativity. Manang Sci Letters. 2019;887–98. doi: 10.5267/j.msl.2019.2.013

[83] Hon AHY, Lui SS. Employee creativity and innovation in organizations. Int J Contempor Hospitality Manag. 2016;28(5):862–85. doi: 10.1108/ijchm-09-2014-0454

[84] Jirásek M, Sudzina F. Big five personality traits and creativity. QIP Journal. 2020;24(3):90–105. doi: 10.12776/qip.v24i3.1509

[85] Amabile TM. A model of creativity and innovation in organizations. Res Organizat Behav. 1988;10.

[86] Amabile TM. The social psychology of creativity: a componential conceptualization. J Person Soc Psychol. 1983;45(2):357–76. doi: 10.1037/0022-3514.45.2.357

[87] Sarooghi H, Libaers D, Burkemper A. Examining the relationship between creativity and innovation: a meta-analysis of organizational, cultural, and environmental factors. J Business Vent. 2015;30(5):714–31. doi: 10.1016/j.jbusvent.2014.12.003

[88] Chaubey A, Sahoo CK, Das KC. Examining the effect of training and employee creativity on organizational innovation: a moderated mediation analysis. Int J Org Anal. 2021;30(2):499–524. doi: 10.1108/ijoa-06-2020-2271

[89] Ghosh K. Developing organizational creativity and innovation. Manag Res Rev. 2015;38(11):1126–48. doi: 10.1108/mrr-01-2014-0017

[90] Shafait Z, Huang J. Exploring the nexus of emotional intelligence and university performance: an investigation through perceived organizational support and innovative work behavior. Psychol Res Behav Manag. 2023;16:4295–313. doi: 10.2147/PRBM.S422194 37900121 PMC10612571

[91] Huang B, Sardeshmukh S, Benson J, Zhu Y. High performance work systems, employee creativity and organizational performance in the education sector. Inter J Human Res Manag. 2022;34(9):1876–905. doi: 10.1080/09585192.2022.2054283

[92] Shafi M, Lei Z, Song X, Sarker MNI. The effects of transformational leadership on employee creativity: moderating role of intrinsic motivation. Asia Pacific Manag Rev. 2020;25(3):166–76. doi: 10.1016/j.apmrv.2019.12.002

[93] Bakker AB, Demerouti E. The Job Demands‐Resources model: state of the art. J Manag Psychol. 2007;22(3):309–28. doi: 10.1108/02683940710733115

[94] Castaneda DI, Cuellar S. Knowledge sharing and innovation: a systematic review. Knowl Process Manag. 2020;27(3):159–73. doi: 10.1002/kpm.1637

[95] Alsaadi FM. Knowledge sharing among academics in higher education institutions in Saudi Arabia. Nova Southeastern University; 2018.

[96] Asad N, Hashmi HBA, Nasir M, Khalid A, Ahmad A. Transformational leadership relationship with employee creativity: the moderating effect of knowledge sharing and mediating effect of creative self-efficacy. Int J Innov Creat Change. 2021;1005–29. doi: 10.53333/ijicc2013/15913

[97] Olan F, Liu S, Neaga I, Alkhuraiji A. How knowledge sharing and business process contribute to organizational performance: using the fsQCA approach. J Business Res. 2016;69(11):5222–7. doi: 10.1016/j.jbusres.2016.04.116

[98] Afandy D, Gunawan A, Stoffers J, Kornarius YP, Caroline A. Improving knowledge-sharing intentions: a study in Indonesian service industries. Sustainability. 2022;14(14):8305. doi: 10.3390/su14148305

[99] Martin JS, Marion R. Higher education leadership roles in knowledge processing. Learn Organ. 2005;12(2):140–51. doi: 10.1108/09696470510583520

[100] Jones G, Sallis E. Knowledge management in education: enhancing learning and education. Routledge; 2013. doi: 10.4324/9780203724910

[101] Kmieciak R. Trust, knowledge sharing, and innovative work behavior: empirical evidence from Poland. EJIM. 2020;24(5):1832–59. doi: 10.1108/ejim-04-2020-0134

[102] Akosile A, Olatokun W. Factors influencing knowledge sharing among academics in Bowen University, Nigeria. J Librarianship Inform Sci. 2019;52(2):410–27. doi: 10.1177/0961000618820926

[103] Cheng MY, Ho JSY, Lau PM. Knowledge sharing in academic institutions: a study of Multimedia University Malaysia. Electron J Knowl Manag. 2009;7(3).

[104] Lee J. The effects of knowledge sharing on individual creativity in higher education institutions: socio-technical view. Administ Sci. 2018;8(2):21. doi: 10.3390/admsci8020021

[105] Kremer H, Villamor I, Aguinis H. Innovation leadership: best-practice recommendations for promoting employee creativity, voice, and knowledge sharing. Business Horizons. 2019;62(1):65–74. doi: 10.1016/j.bushor.2018.08.010

[106] Belso-Martinez JA, Diez-Vial I. Firm’s strategic choices and network knowledge dynamics: how do they affect innovation?. JKM. 2018;22(1):1–20. doi: 10.1108/jkm-12-2016-0524

[107] Elamin AM, Aldabbas H, Ahmed AZE, Abdullah AN. Employee engagement and innovative work behavior: the mediating role of knowledge-sharing behavior in the United Arab Emirates (UAE) Service Context. Administ Sci. 2024;14(9):232. doi: 10.3390/admsci14090232

[108] Faris Hussain M, Hanifah H, Vafaei-Zadeh A, Abdul Halim H. Determinants of innovative work behavior and job performance: moderating role of knowledge sharing. Int J Innov Technol Management. 2022;20(01). doi: 10.1142/s0219877022500377

[109] Thneibat MM. The impact of high commitment work practices on radical innovation: innovative work behaviour and knowledge sharing as mediators. Int J Prod Perf Manag. 2024;73(7):2329–63. doi: 10.1108/ijppm-01-2023-0036

[110] Amin A, Basri S, Hassan MF, Rehman M. Occupational stress, knowledge sharing and GSD communication barriers as predictors of software engineer’s creativity. In: 2011 IEEE International conference on industrial engineering and engineering management. IEEE; 2011.

[111] Kim W, Park J. Examining structural relationships between work engagement, organizational procedural justice, knowledge sharing, and innovative work behavior for sustainable organizations. Sustainability. 2017;9(2):205. doi: 10.3390/su9020205

[112] Chen M, Zada M, Khan J, Saba NU. How does servant leadership influences creativity? Enhancing Employee creativity via creative process engagement and knowledge sharing. Front Psychol. 2022;13:947092. doi: 10.3389/fpsyg.2022.947092 35846716 PMC9284035

[113] Despita CW, Yuliani KF, Tanuwijaya J. Pengaruh high performance work systems terhadap community participation melalui feeling trusted dan knowledge sharing behavior. J MAS. 2022;7(1):299. doi: 10.33087/jmas.v7i1.398

[114] Almadana AV, Suharnomo S, Perdhana MS. Can generational differences and feeling trusted improve knowledge-sharing behavior? Consequences of high-performance work systems. J Workplace Learn. 2021;34(2):200–14. doi: 10.1108/jwl-05-2021-0058

[115] Abbasi SG, Shabbir MS, Abbas M, Tahir MS. HPWS and knowledge sharing behavior: the role of psychological empowerment and organizational identification in public sector banks. J Public Affairs. 2020;21(3). doi: 10.1002/pa.2512

[116] Chuang C-H, Jackson SE, Jiang Y. Can knowledge-intensive teamwork be managed? Examining the roles of HRM systems, leadership, and tacit knowledge. J Manag. 2013;42(2):524–54. doi: 10.1177/0149206313478189

[117] Bhatti SH, Zakariya R, Vrontis D, Santoro G, Christofi M. High-performance work systems, innovation and knowledge sharing: an empirical analysis in the context of project-based organizations. Empl Relat Int J. 2020;43(2):438–58. doi: 10.1108/er-10-2019-0403

[118] Bell E, Harley B, Bryman A. Business research methods. Oxford University Press; 2022.

[119] Alnehabi M, Al-Mekhlafi A-BA. The Association between corporate social responsibility, employee performance, and turnover intention moderated by organizational identification and commitment. Sustainability. 2023;15(19):14202. doi: 10.3390/su151914202

[120] Olowoye OA. Relationship between perceived funding level and service quality for employees in south western nigeria universities. Delaware: Wilmington University; 2020.

[121] Krejcie RV, Morgan DW. Determining sample size for research activities. Edu Psychol Measure. 1970;30(3):607–10. doi: 10.1177/001316447003000308

[122] Hoo WC, Khee KH, Wolor CW, Teck TS, Toh JS. Determinants of intention to use buy now pay later (BNPL). J Lifestyle SDGs Rev. 2024;5(1):e02698. doi: 10.47172/2965-730x.sdgsreview.v5.n01.pe02698

[123] Hair JF, Black WC, Babin BJ, Anderson RE, Tatham RJ. Multivariate data analysis. New Jersey: Pearson Education; 2010.

[124] Creswell JW, Creswell JD. Research design: qualitative, quantitative, and mixed methods approaches. Sage publications; 2017.

[125] Saunders M, Lewis P, Thornhill A. Research methods for business students. Pearson Education; 2009.

[126] Umrani WA, Siyal S, Al Riyami S, Memon MA, Siyal AW. Inclusive leadership and innovative work behaviours: social exchange perspective. Curr Psychol. 2024;43(30):24774–88. doi: 10.1007/s12144-024-06192-1

[127] Ng PML, Wut TM, Lo MF. Enhancing knowledge sharing behaviour in building academics’ career capital in higher education: the mediating role of innovative climate. Tech Know Learn. 2022;29(1):91–111. doi: 10.1007/s10758-022-09633-7

[128] Pu R, Dong RK, Jiang S. Toward the education for sustainable development (ESD): Digital leadership and knowledge-sharing behavior on the higher education institutional change. Educ Inf Technol. 2024;30(8):10567–89. doi: 10.1007/s10639-024-13247-0

[129] Lohmöller JB. Latent variable path modeling with partial least squares. Springer Science and Business Media; 2013.

[130] Götz O, Liehr-Gobbers K, Krafft M. Evaluation of structural equation models using the partial least squares (PLS) approach. In: Handbook of partial least squares. Springer Berlin Heidelberg; 2009. 691–711. doi: 10.1007/978-3-540-32827-8_30

[131] Morgan CW. University staff; creativity and innovation in higher education: Arizona State University; 2020.

[132] Scott SG, Bruce RA. Determinants of innovative behavior: a path model of individual innovation in the workplace. Acad Manag J. 1994;37(3):580–607. doi: 10.2307/256701

[133] Wang H, Chen X, Wang H, Xie M. Employee innovative behavior and workplace wellbeing: Leader support for innovation and coworker ostracism as mediators. Front Psychol. 2022;13:1014195. doi: 10.3389/fpsyg.2022.1014195 36524195 PMC9744940

[134] Wang Y, Zhu L, Jin X. The effect of a high-performance work system on organizational innovation performance: the mediating effect of employees’ intrinsic motivation and the moderating effect of person–organization fit. Systems. 2024;12(7):230. doi: 10.3390/systems12070230

[135] Xu Z, Suntrayuth S. Innovative work behavior in high-tech enterprises: chain intermediary effect of psychological safety and knowledge sharing. Front Psychol. 2022;13:1017121. doi: 10.3389/fpsyg.2022.1017121 36353082 PMC9637621

[136] Anderson JC, Gerbing DW. Structural equation modeling in practice: a review and recommended two-step approach. Psychol Bulletin. 1988;103(3):411–23. doi: 10.1037/0033-2909.103.3.411

[137] Hair J, Hollingsworth CL, Randolph AB, Chong AYL. An updated and expanded assessment of PLS-SEM in information systems research. IMDS. 2017;117(3):442–58. doi: 10.1108/imds-04-2016-0130

[138] Yesuf YM, Getahun DA, Debas AT. Factors affecting “employees’ creativity”: the mediating role of intrinsic motivation. J Innov Entrep. 2023;12(1). doi: 10.1186/s13731-023-00299-8

[139] Fornell C, Larcker DF. Structural equation models with unobservable variables and measurement error: algebra and statistics. Los Angeles, CA: Sage Publications; 1981.

[140] Henseler J, Ringle CM, Sarstedt M. A new criterion for assessing discriminant validity in variance-based structural equation modeling. J Acad Mark Sci. 2014;43(1):115–35. doi: 10.1007/s11747-014-0403-8

[141] Falk RF, Miller NB. A primer for soft modeling. University of Akron Press; 1992.

[142] Baron RM, Kenny DA. The moderator-mediator variable distinction in social psychological research: conceptual, strategic, and statistical considerations. J Pers Soc Psychol. 1986;51(6):1173–82. doi: 10.1037//0022-3514.51.6.1173 3806354

[143] F. Hair Jr J, Sarstedt M, Hopkins L, G. Kuppelwieser V. Partial least squares structural equation modeling (PLS-SEM). Europ Business Rev. 2014;26(2):106–21. doi: 10.1108/ebr-10-2013-0128

[144] Arshad B, Hassan H, Azam A. The impact of employees’ experience of high-performance work systems on innovative behavior in professional service firms. Front Psychol. 2024;14:1324474. doi: 10.3389/fpsyg.2023.1324474 38259570 PMC10800686

[145] Farrukh M, Khan MS, Raza A, Shahzad IA. Influence of high-performance work systems on intrapreneurial behavior. J Sci Tech Pol Manag. 2021;12(4):609–26. doi: 10.1108/jstpm-05-2020-0086

[146] Imran R, Al-Ansi KSH. High performance work system, job engagement and innovative work behavior: An exploration in Omani context. In: Proceedings of the 2019 2nd International Conference on Computers in Management and Business, 2019.

[147] Abdillah MR, Asfar A. High performance work system (HPWS) and teacher’s innovative work behavior: the role of proactive behavior. Sains Organisasi. 2023;2(2):129–42. doi: 10.55356/so.v2i2.56

[148] Van De Voorde K, Paauwe J, Van Veldhoven M. Employee well‐being and the HRM–organizational performance relationship: a review of quantitative studies. Int J Manag Rev. 2011;14(4):391–407. doi: 10.1111/j.1468-2370.2011.00322.x

[149] Kroon B, van de Voorde K, van Veldhoven M. Cross‐level effects of high‐performance work practices on burnout. Personnel Rev. 2009;38(5):509–25. doi: 10.1108/00483480910978027

[150] Purnama Sari NF, Wahyuni S. The role of mediation and moderation in the sharing of knowledge and work centrality effects of employee creativity on innovative work behavior. Int J Econ Business Manag Res. 2023;07(06):44–56. doi: 10.51505/ijebmr.2023.7604

[151] Masianoga ES, Chakauya L. A framework for employee creative and innovative behaviour idea journey. Open J Business Manag. 2023;11(03):839–50. doi: 10.4236/ojbm.2023.113045

[152] Jolaee A, Md Nor K, Khani N, Md Yusoff R. Factors affecting knowledge sharing intention among academic staff. Inter J Educ Manag. 2014;28(4):413–31. doi: 10.1108/ijem-03-2013-0041

[153] Kim TT, Lee G. Hospitality employee knowledge-sharing behaviors in the relationship between goal orientations and service innovative behavior. Inter J Hospit Manag. 2013;34:324–37. doi: 10.1016/j.ijhm.2013.04.009

[154] Alpkan L, Bulut C, Gunday G, Ulusoy G, Kilic K. Organizational support for intrapreneurship and its interaction with human capital to enhance innovative performance. Manag Decision. 2010;48(5):732–55. doi: 10.1108/00251741011043902

[155] Dobni CB. The relationship between innovation orientation and organisational performance. Int J Innov Learn. 2011;10(3):226. doi: 10.1504/ijil.2011.042078

[156] Rai A, Ghosh P, Chauhan R, Mehta NK. Influence of job characteristics on engagement: does support at work act as moderator?. Int J Sociol Soc Pol. 2017;37(1/2):86–105. doi: 10.1108/ijssp-10-2015-0106

