# Supplementary material for: High performance work system and innovative work behaviour: A moderated mediation analysis of knowledge sharing and employee creativity in Nigerian higher education institutions
Source: PLoS One. 2025 Dec 31;20(12):e0338031. doi: 10.1371/journal.pone.0338031 (PMC12755793; doi:10.1371/journal.pone.0338031)
Supplement: S1 File — (DOCX) [file pone.0338031.s001.docx]

Appendix A

Table A1. Research instruments

| Variable | Item | Reference |
| --- | --- | --- |
| Innovative work behavior | IWB1. I search out new technologies, processes, techniques, and/or product ideas.  IWB 2. I often generate creative ideas.  IWB 3. I often promote and champion ideas to others.  IWB 4. I investigate and secure funds needed to implement new ideas.  IWB 5. I develop adequate plans and schedules for the implementation of new ideas.  IWB 6. Overall I am innovative. | Wang, Chen [156] |
| Employee Creativity | EC1. I suggest new ways to achieve goals and objectives.  EC2. I come up with new and practical ideas to improve performance.  EC3. I search out new technologies, processes, techniques, and/or product ideas.  EC4. I suggest new ways to increase quality.  EC5. I have a good source of creative ideas.  EC6. I am not afraid to take risks.  EC7. I promote and champion ideas to others.  EC8. I exhibit creativity on the job when given the opportunity.  EC9. I develop adequate plans and schedules for the implementation of new ideas.  EC10. I often have new and innovative ideas.  EC11. I come up with creative solutions to problems.  EC12. I often have a fresh approach to problems.  EC13. I suggest new ways of performing work tasks.‎ | Nasifoglu Elidemir, Ozturen [2] |
| High-Performance Work System | ‎HPWS1. This university offers training to improve the interpersonal skills of employees. ‎  ‎ HPWS 2. New employees undergo extensive orientation training in order to learn the values and ‎culture of this subsidiary and/or its American parent company. ‎  ‎ HPWS 3. Many of this subsidiary’s employees are moved through a series of different job ‎assignments in order to prepare them for future assignments. ‎  ‎ HPWS 4. This subsidiary devotes considerable resources to manager training and development. ‎  ‎ HPWS 5. We do a great deal of cross-training, so that managers are familiar with different jobs and ‎can fill in for others when necessary. ‎  ‎ HPWS 6. The employee selection process is very rigorous in this subsidiary (e.g., use of tests, ‎aptitude test, interviews, etc.).  ‎ HPWS 7. There is advance planning as to which of this subsidiary’s current employees will be ‎transferred or promoted when there is a job vacancy. ‎  ‎ HPWS 8. An employee’s job performance is appraised, to a significant extent, on how well he or ‎she follows orders and company procedures and rules. ‎  ‎ HPWS9. We strive to keep a large salary difference between high and low performers in the same ‎position. ‎  ‎ HPWS10. An employee’s pay is closely tied to individual or group performance in this subsidiary. ‎‎  HPWS11. Employees often work in self-directed teams. ‎  ‎HPWS12.This subsidiary extensively shares its financial and/or performance data with its ‎employees.‎ | ‎Wang et al. (2024)‎ |
| Knowledge sharing | ‎KS1.In my daily work, I take the initiative to impart academic knowledge to colleagues. ‎  ‎ KS 2. I share useful work experience and ideas with everyone. ‎  ‎ KS 3. After learning new knowledge useful for work, I share it so that more people can learn it. ‎  ‎ KS 4. At work, I take out my knowledge to share with more people. ‎  ‎ KS 5. I actively use the university's existing information technology to share my knowledge. ‎  ‎ KS 6. As long as other colleagues need it, I always say all my know and say it without reserve.‎ | Xu and Suntrayuth [134] |
